# Multi-Bioactivity of Protein Digests and Peptides from Oat (*Avena sativa* L.) Kernels in the Prevention of the Cardiometabolic Syndrome

**DOI:** 10.3390/molecules27227907

**Published:** 2022-11-15

**Authors:** Małgorzata Darewicz, Monika Pliszka, Justyna Borawska-Dziadkiewicz, Piotr Minkiewicz, Anna Iwaniak

**Affiliations:** Department of Food Biochemistry, Faculty of Food Science, University of Warmia and Mazury in Olsztyn, Pl. Cieszynski 1, 10-726 Olsztyn, Poland

**Keywords:** bioactive peptides, BIOPEP-UWM database, digestion, cardiometabolic syndrome, oat kernels, absorption

## Abstract

The aim of this study was to characterize the digests and peptides derived from oat kernel proteins in terms of their major enzyme inhibitory activities related to the prevention of cardiometabolic syndrome. It also entailed the characteristics of antioxidant bioactivity of the analyzed material. The study was carried out using coupled in silico and in vitro methods. The additional goal was to investigate whether identified peptides can pervade Caco-2 cells. Based on the results of bioinformatic analysis, it was found that the selected oat proteins may be a potential source of 107 peptides with DPP-IV and/or ACE inhibitory and/or antioxidant activity. The duodenal digest of oat kernels revealed multiple activities. It inhibited the activities of the following enzymes: DPP-IV (IC_50_ = 0.51 vs. 10.82 mg/mL of the intact protein), α-glucosidase (IC_50_ = 1.55 vs. 25.20 mg/mL), and ACE (IC_50_ = 0.82 vs. 34.52 mg/mL). The DPPH^•^ scavenging activity was 35.7% vs. 7.93% that of the intact protein. After in silico digestion of oat proteins, 24 peptides were selected for identification using LC-Q-TOF-MS/MS. Among them, 13 sequences were successfully identified. One of them, i.e., VW peptide, exhibited triple activities, i.e., DPP-IV and ACE inhibitory and DPPH^•^ scavenging activity. The multifunctional peptides: PW, TF, VF, and VW, were identified in the basolateral samples after transport experiments. Both in silico and in vitro analyses demonstrated that oat kernel proteins were the abundant sources of bioactive digests and peptides to be used in a diet for patients suffering from cardiometabolic syndrome.

## 1. Introduction

The beginning of the 21st century has brought new insight into conventional food that is perceived as a source of not only nutrients but also biologically-active compounds necessary to maintain homeostasis and ensure proper body functions. These compounds include food proteins, and biologically active peptides (BAPs) derived from foods that are built mainly of 2-20 amino acid residues [1]. BAPs can be released during: (i) conventional enzymatic proteolysis; (ii) fermentation processes; (iii) coupled use of both these methods; and (iv) technological processes including, e.g., high-pressure treatment [2]. Biopeptides can also be synthesized in chemical reactions or obtained through the expression of appropriate genes [3]. Considering the importance of BAPs to dietary prophylaxis, Minkiewicz et al. [4] have proposed a concept of a food peptidome as a pool of all peptides derived from proteins of raw materials and food products. The exploitation of BAPs represents a new strategy for the treatment of diet-related lifestyle diseases, with cardiometabolic syndrome (CMS) as an example. Cardiometabolic syndrome (also referred to as the metabolic syndrome X, insulin resistance syndrome, or Raeven’s syndrome) is a term legalized by the World Health Organization (WHO) and included in the International Statistical Classification of Diseases and Related Health Problems as a non-communicable disease caused by metabolic disorders [5]. CMS represents the clustering of interconnected metabolic risk factors, which results in chronic subclinical hypertension, glucose intolerance, and inflammation [6]. The consequences of risk posed by the individual disease entities sum up and augment, which increases the risk of adverse complications. The CMS affects ca. 36% of women and ca. 38% of men in the European population. To date, there is no single effective drug treatment that would affect all of CMS components at the same time [7]. Therefore, one of the trends in the studies into the bioactive food protein hydrolysates and BAPs entails their multifunctional properties, which could be harnessed in some aspects of CMS prevention and even features treatment when considering their role as inhibitors of: dipeptidyl peptidase IV (DPP-IV) [EC 3.4.14.5], α-glucosidase [EC 3.2.1.20], angiotensin I-converting enzyme (ACE) [EC 3.4.15.1], and as antioxidative peptides. According to Iwaniak and Mogut [8], peptides exhibiting more than one biological activity can be more powerful against lifestyle diseases than those eliciting only one effect. Peptides revealing dual activities, i.e., antioxidative and ACE-inhibitory ones, were isolated by Garcia-Mora et al. [9] from lentil proteins hydrolyzed using Savinase^®^. The peptide inhibitors of both ACE and DPP-IV were obtained from Antarctic krill proteins hydrolyzed using Corolase PP^®^ [10], whereas multifunctional peptide inhibitors of DPP-IV, α-glucosidase, and ACE, additionally exhibiting the antioxidative activity, were obtained from hen egg yolk proteins [11].

Oats are cereals that rank sixth, after wheat, rice, maize, barley, and sorghum, in the global production of grains [12]. Dietary interventions in CMS prevention involving oats inclusion have so far been mainly driven by the presence of β-glucan, which is responsible for suppressing lipid absorption and the appearance of hunger sensation between meals or increasing insulin sensitivity [13]. Investigations addressing the biological activity of peptides ‘hidden’ in oat protein sequences have so far been involved in in silico analyses [14] or have concerned synthetic peptides whose sequences were predicted based on these analyses [15]. Other studies have concerned peptides obtained upon enzymatic hydrolysis with various single enzymes, e.g., trypsin [EC 3.4.21.4], thermolysin [EC 3.4.24.27], or with commercial enzymatic preparations [16,17,18]. In turn, in the study by Dugardin et al. [19], a hydrolyzed oat protein preparation was obtained using an in vitro digestion model. However, this model was based on Versantvoort et al. protocol [20] which was applied in a very limited number of studies.

To the best of our knowledge, no studies have yet been undertaken concerning the dietary CMS prophylaxis related to the release of biologically active multifunctional hydrolysates and peptides from oat kernel proteins. When searching for CMS-preventive peptides, it is essential to find peptides that are simultaneously involved in the prevention of more than one mechanism of CMS. Therefore, our study aimed to investigate the potential of oat kernel protein digests/peptides to modulate the key enzymes in CMS pathogenesis and their antioxidative activity. The first objective was to establish whether oat kernel protein fractions contain peptide sequences that can inhibit DPP-IV, α-glucosidase, ACE, and sequences of antioxidative peptides and whether these peptides can be released during simulated digestion of oat proteins. The second objective was to evaluate the inhibitory activity against DPP-IV, ACE, α-glucosidase, and antioxidative activity of the oat digests and peptides obtained. Moreover, the possibilities of the transepithelial transport of oat-released multifunctional peptides were investigated using the Caco-2 monolayer.

## 2. Results and Discussion

### 2.1. Oat Kernel Proteins In Vitro Human-Like Gastrointestinal Digestion

**Figure 1:**
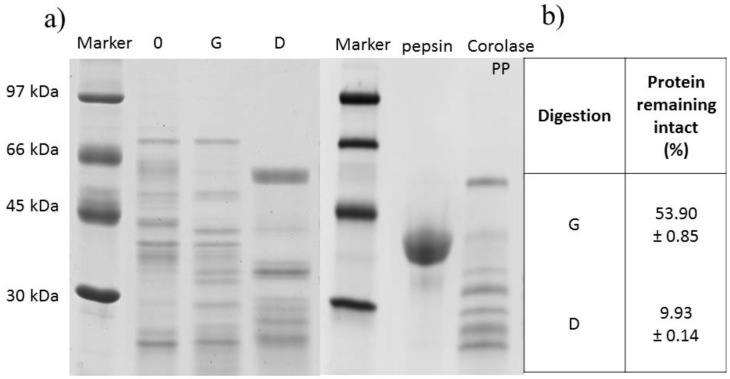
Analysis of oat kernel proteins after in vitro human-like gastrointestinal digestion. (**a**) Electrophoretic separation (SDS-PAGE) of digests of oat kernel proteins and digestive enzymes. (**b**) The percentage of protein remaining intact after digestion of oat kernel proteins. Mass marker (30–97 kDa), 0—intact sample, G—digest after 2 h gastric phase, D—digest after 2 h duodenal phase (see Materials and Methods).

Physicochemical properties and molecular weight of bioactive peptides may influence their bioactivity or target binding mode. According to the scientific data, the dehusked kernels are significantly higher in protein and lower in crude fiber; therefore, naked grains were used in our study to obtain a higher content of this nutrient in the resulting digests [17]. The protein content of oat kernels used in the experiment reached 15% and was comparable with values reported by other authors, i.e., from 15% to 20% [21]. In our study, oat kernel proteins were digested using the INFOGEST static in vitro digestion protocol. Sanchón et al. [22], who used human digests for comparison, stated that the INFOGEST model constituted a good approximation to the physiological gastrointestinal digestion of milk proteins. Moreover, according to Sousa et al. [23], due to the simplicity of the INFOGEST model helping to predict the results of in vivo digestion, it can be used to digest proteins from animal and plant sources. After two hours of oat protein digestion with pepsin, the content of protein remaining intact was at 53.90%, which indicates that almost half of the proteins were digested into smaller fragments (Figure 1b). The continued digestion with Corolase PP at the duodenal phase contributed to a decreased content of non-digested protein to 9.93%. Results of these determinations point to a high degree of defragmentation of oat kernel proteins after the duodenal phase. Furthermore, electrophoretic separations of the digests were performed in a polyacrylamide gel in the presence of SDS to enable monitoring the oat protein digestion (Figure 1a). After digestion with pepsin (G digest), the intensity of the bands staining in the gel changed slightly respective to the undigested sample. In contrast, significant differences were noticed in the number, arrangement, and the bands intensity staining, or even their complete decay after the digestion with pepsin and Corolase PP (D digest) compared to the digestion with pepsin only (G digest).

The analysis of electropherograms allowed concluding that the largest group of proteins in the intact sample was represented by protein fractions with molecular weights (MW) ranging from 30 to 67 kDa, although the staining of some fractions having MW above 67 kDa and below 30 kDa could be observed as well. These results and findings published by other authors [24] enabled identifying bands on electropherograms with a molecular weight corresponding to globulin 12S and its subunits with MW of ca. 30–42 kDa and 19–25 kDa. Moreover, other bands stained at ca. 55 kDa, which probably corresponded to globulin 7S, were also detected. Schalk et al. [24] concluded that oat kernel globulins showed certain similarities to the globulins of legume seeds and other dicotyledonous plants. With the in vitro human-like gastrointestinal digestion proceeding, bands with molecular weights above 30 kDa disappeared, whereas new bands appeared that had lower molecular weights and were products of oat kernel protein defragmentation. The digestion at the duodenal phase was much more advanced than at the gastric phase. Similar observations were made in other experiments conducted with the INFOGEST in vitro digestion model methods [22,23,25,26]. 

### 2.2. Dipeptidyl Peptidase IV Inhibitory Activity

**Figure 2:**
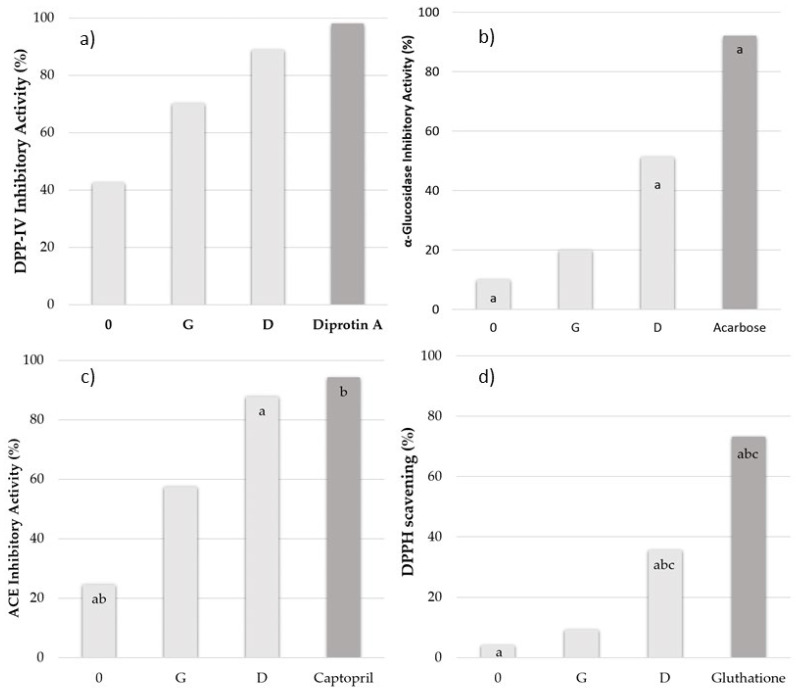
Bioactivity of digested oat kernel proteins expressed as % of inhibitory activity: (**a**) DPP-IV inhibitory, (**b**) α-glucosidase inhibitory, (**c**) ACE inhibitory and (**d**) antioxidant activities; where 0—intact sample, G—sample after 2 h gastric phase, D—sample after 2 h duodenal phase (see Section 3). Value means ± SD of three determinations. The bars denoted with the same letter (a, b, c) differ statistically (*p* < 0.05).

Inhibitors of dipeptidyl peptidase IV, i.e., an enzyme responsible for the proteolysis of incretins (insulinotropic intestinal enzymes: glucagon-like peptide 1 and glucose-dependent insulinotropic polypeptide), play an important role in regulating glucose homeostasis. Synthetic DPP-IV inhibitors, i.e., gliptins (e.g., sitagliptin, saxagliptin, vildagliptin), are well tolerated; however, their undesirable side effects are observed in some patients [13]. Inhibitory activity of oat kernel digests obtained due to the action of Corolase PP and/or pepsin against human recombinant DPP-IV was determined in the present experiment (see Figure 2a). As the hydrolysis of oat kernel proteins progressed, their DPP-IV inhibitory activity steadily increased from 42.55 ± 0.05% (intact sample) through 70.20 ± 0.05% (gastric sample; G) to 89.51 ± 0.12% (duodenal sample; D). The potential of oat kernel digests to inhibit the DPP-IV increased approximately to the same extent in the gastric and duodenal phases of digestion. It is worth emphasizing that the sample after the duodenal stage of digestion was almost as effective (89%) as the DPP-IV inhibitor as the standard diprotin A (97%), a compound that suppresses the degradation of incretins by blocking DPP-IV action.

The intact sample showed an IC_50_ of 10.82 mg/mL (Table 1). The inhibitory activity after the gastric phase increased compared to the intact sample, as indicated by IC_50_ equal to 4.23 mg/mL. The highest DPP-IV inhibitory activity was demonstrated for the duodenal phase digest, which suppressed the enzyme activity with an IC_50_ equal to 0.51 mg/mL.

These results are in line with those described in the previous section regarding protein content after digestion and electrophoretic separation. The gastric phase with the upcoming duodenal one seems to offer a means to produce peptides with relatively high potency to inhibit DPP-IV activity, which was also confirmed by their IC_50_ values. The shorter peptides released at the terminal phase of digestion are more effective inhibitors than these, which contain more amino acid residues [27]. The last decade has brought results of some investigations into the DPP-IV inhibitory activity of proteins from milk, fish, wheat gluten, beans, and eggs after enzymatic hydrolysis, fermentation, or treatments with physical and chemical methods [14,27]. Nongonierma et al. [28], who used Debitrase HYW20 and Corolase PP preparations, obtained wheat gluten hydrolysates with DPP-IV inhibitory activity (IC_50_ = 0.33 mg/mL). The activity of DPP-IV was also inhibited by quinoa hydrolysate (IC_50_ = 0.88 mg/mL) [29] and by soybean protein hydrolysates after simulated digestion (IC_50_ = 1.49 mg/mL), of which the most active were these with molecular weights of 5–10 kDa [30]. It is worth mentioning that the number of studies related to the inhibition of DPP-IV activity by plant protein hydrolysates is rather rare, while the number of experiments involving human DPP-IV, as it was presented in this study, is even more unique. To the best of our knowledge, no studies concerning the DPP-IV-inhibiting potential of oat hydrolysates were carried out with human DPP-IV. Dugardin et al. [19] produced oat hydrolysates with DPP-IV inhibitory activity using DPP-IV from porcine kidney. Based on IC_50_ values ranging from 0.75 to 0.83 mg/mL, these hydrolysates can be found as less potent than those obtained in our experiment. In turn, human DPP-IV was applied by Lammi et al. [31], who hydrolyzed soybean proteins with pepsin and achieved enzyme inhibition increase by ca. 30%.

To summarize, according to Li et al. [32], restoring incretin activity by digests/peptides derived from oat proteins without undesirable symptoms can offer new opportunities for modern prophylaxis or even therapy to improve the metabolic homeostasis of glucose. Our results seem to confirm that this prophylaxis can be supported by oat kernel protein digests, which exhibit a high in vitro potential to suppress human DPP-IV activity.

### 2.3. α-Glucosidase Inhibitory Activity

Glucosidase inhibitors impede the uptake of glucose from a dietary complex of carbohydrates ultimately into the bloodstream, thereby effectively reducing postprandial hyperglycemia. Inhibitors of α-glucosidase are commonly used as orally-administered drugs in the pharmacological therapy of diabetes and postprandial hyperglycemia [30]. They include synthetic inhibitors, e.g., acarbose, miglitol, voglibose, and emiglitat. Nevertheless, similarly to DPP-IV and angiotensin convertase inhibitors, the prolonged use of these oral drugs can cause side effects, e.g., bloating, abdominal cramps, vomiting, and diarrhea [13]. For this reason, greater attention has been paid in recent years to the food products being sources of compounds inhibiting α-glucosidase activity from aiding the therapy/prophylaxis of diabetic patients. In this context, worthy of consideration are oat protein digests also exhibiting α-glucosidase inhibitory properties as supporting and auxiliary factors of DPP-IV inhibitors. Therefore, the characterization of the potentially antidiabetic properties of oat kernel protein digests was completed with determinations of their α-glucosidase inhibitory activity. Before digestion, the oat kernel extracts showed a slight α-glucosidase inhibitory activity. Similarly, slight α-glucosidase inhibiting activities of oat digests were observed compared to the control sample. After the gastric phase, the extracts increased α-glucosidase inhibition twice, from 10.04 ± 0.04% to 19.95 ± 0.12%. In turn, after duodenal digestion, the inhibition of α-glucosidase boosted to 51.29 ± 0.06% (see Figure 2b). The IC_50_ values presented in Table 1 indicate the possibility of α-glucosidase activity inhibition during in vitro digestion of oat kernel proteins. The IC_50_ values obtained in the particular digestion phases decreased from 10.35 ± 0.03 mg/mL in the gastric phase to 1.55 ± 0.04 mg/mL in the duodenal phase. Results presented in Figure 2b and Table 1 show that oat protein digest after the duodenal phase of digestion is a considerably weaker inhibitor of α-glucosidase than acarbose. Results of determinations of α-glucosidase inhibitory activity of oat protein digests are comparable to literature data concerning the experiments on plant proteins. A similar potential to inhibit the activity of α-glucosidase (i.e., from 36.3 to 50.1%) was demonstrated for common bean proteins after simulated digestion [33]. Hydrolysates obtained with the use of Alcalase of brewer’s spent grain proteins were presented to inhibit the activity of the enzyme by 56%. Higher activity was shown for black bean proteins (66.1%) also hydrolyzed with Alcalase [34]. Furthermore, proteins of common bean with the so-called hard-to-cook defect, hydrolyzed with Alcalase and bromelain, showed over 50% α-glucosidase inhibitory activity after 120 min of hydrolysis, with the highest activity (76.4%) observed for a fraction of the hydrolysate obtained using Alcalase with MW < 1 kDa [35].

Taking into account our results as well as literature data concerning the oral peptide drugs acting as α-glucosidase inhibitors, it can be concluded that oat proteins are valuable food nutrients generating α-glucosidase inhibiting effect and, thus, should be considered as food promoted in CMS prevention.

### 2.4. Angiotensin I-Converting Enzyme Inhibitory Activity

The compounds acting as ACE inhibitors can be used in the prophylaxis of cardiovascular diseases. Due to the associated side effects of synthetic ACE inhibitors, e.g., captopril, enalapril, and fosinopril, alternative sources of ACE inhibitors are being sought, including proteins of plant-derived foods [36]. Results of analyses of the angiotensin-I converting enzyme inhibitory activity of oat kernel protein digests are shown in Figure 2c. ACE inhibitory activity steadily increased as oat protein digestion proceeded. The ability of the in vitro digests derived from oat kernel proteins to inhibit the ACE ranged from 24.49 ± 0.65% through 57.34 ± 2.34% to 87.76 ± 0.65%. The highest inhibitory activity was demonstrated for the digest after two digestion phases, likewise the highest half maximal inhibitory concentration—i.e., for the sample after the duodenal phase (IC_50_ =0.82 mg/mL; see Table 1). The digest obtained after the gastric phase was less active, with an IC_50_ equal to 8.89 mg/mL. At the same time, unlike the DPP-IV inhibitory activity, oat protein D-phase digest was a bit less active when compared to the synthetic inhibitor, i.e., captopril activity. Our findings are in line with the bioactivity of other food protein hydrolysates reported for lentil [37], Adzuki bean [38], or hen egg albumin [11] protein hydrolysates and even relatively higher to these determined by Darewicz et al. [39] and Borawska et al. [25] for carp and salmon in vitro digests. All the aforementioned studies demonstrated that peptide fractions having low molecular weights exhibited a higher ACE-inhibitory activity than the high-molecular-weight ones. Results obtained in the present study also correspond with findings reported by Akillioğlu and Karakaya for legume proteins [40], according to which the IC_50_ values of in vitro common bean digests were similar to these obtained in our study, i.e., 0.78–0.83 mg/mL, whereas those of pinto beans were lower, i.e., 0.69 mg/mL.

To recapitulate, oat protein digests reveal very good properties to inhibit the activity of the angiotensin-I converting enzyme, which may offer a good dietary alternative for patients suffering from cardiovascular diseases. 

### 2.5. Antioxidative Activity 

Digested oat kernel proteins were characterized for their antioxidative activity, which was expressed as the ability to scavenge free DPPH radical (see Figure 2d; Table 1). The antioxidative activity determined with the DPPH^•^ method increased in the successive stages of digestion. After 2 h of gastric digestion, it was higher than in the intact sample (9.4% and 9.91 ± 0.15 µM Trolox/mg vs. 4.17 ± 0.23% and 7.93 ± 0.07 µM). The duodenal phase of digestion contributed to the significant increase in the DPPH radical scavenging ability of the digest, with the highest activity reaching 35.70 ± 0.54% and 33.93 ± 0.26 µM Trolox/mg of sample. The free DPPH radical quenching determined for the duodenal sample was, however, significantly lesser than that determined for glutathione, which is an antioxidant used as a proton donor. Due to the low content of non-hydrolyzed protein in the analyzed samples, it can be concluded that, after in vitro digestion, the oat kernel protein digests contained mainly low-molecular-weight fragments and free amino acids. Smaller peptides are released after simulated in vitro digestion in the group in which both hydrophilic and hydrophobic amino acids may appear. This could be the likely reason for the low free DPPH radical scavenging ability of intestinal oat digests compared to glutathione. The simulated digestion can cause the appearance of not only hydrophobic molecules but also hydrophilic peptides and amino acids, which cannot enter into reactions with the lipophilic soluble DPPH radical [41]. The in silico part of the study shows that although 60% of the peptides encrypted in oat protein sequences and released after digestion (see Table S2) were poorly soluble, 40% of them were characterized by good solubility.

Esteve et al. [42] demonstrated that short-chain peptides obtained after the hydrolysis of extracts of proteins from a waste product after olive oil production with Alcalase exhibited a significantly higher antioxidative potential compared to the high-molecular-weight peptides. Moreover, Ngoh and Gan [43] proved that, among a few fractions of pinto bean protein hydrolysates obtained using membrane ultrafiltration, the highest antioxidative activity was shown by the peptide fraction with a molecular weight lower than 3 kDa. In turn, Park et al. [44] demonstrated that the low-molecular-weight fraction (<3 kDa) of skimmed soybean protein hydrolysate could also be a strong antioxidant. The mechanisms of the antioxidative effect of peptides have not been fully recognized yet, although they were proven to act as scavengers of free radicals, inhibitors of lipid oxidation, or metal ion chelators [45]. Results of this part of the experiment indicate that the oat digests contain peptides and amino acids that can scavenge free radicals in non-polar solutions to a certain degree. 

### 2.6. Identification of Cardiometabolic Syndrome-Preventive Peptides

Proteolysis of oat kernel proteins with pepsin, trypsin, and chymotrypsin using the BIOPEP-UWM ‘Enzyme Action(s)’ function resulted in the potential release of 107 sequences of peptide inhibitors of CMS enzymes and antioxidative peptides (see Table S2). More than 60% of the peptides presented in Table S2 were poorly soluble. Apart from the functional properties of the protein, such as emulsifying, foaming, and water-holding capacity, solubility, along with amphiphilicity, is found as a crucial factor in the biological activity of proteins and peptides. It results from the presence of motifs mostly containing amino acids with a hydrophobic nature [13,46]. 

After in silico proteolysis of oat proteins, some peptides were selected for identification. The criterion of the selection was the score value higher than 0.75 computed with PeptideRanker. The score value estimates the likelihood of the peptide being bioactive [47]. It led to ranking 24 sequences (see Table 2) whose score value was from 1.00 to 0.79 (for MF and PR dipeptides, respectively). The sequences with PeptideRanker score values up to 0.69 were rejected from identification analysis. However, noteworthy is that the PeptideRanker is a screening tool suitable to show the potential of the sequence to act as a bioactive agent with no indication of its particular bioactivity [48]. Furthermore, the *p*-values (values equal to 0.01 and less), describing the potential interactions with DPP-IV and ACE, were calculated for all selected sequences using Pepsite2. All selected 24 peptides were significantly interacting with DPP-IV and ACE (*p*-value ˂ 0.05). DPP-IV and ACE comprise some pockets with binding sites where peptides (ligands) can “fit” [27]. In the case of ACE inhibitors, the lowest *p*-value was obtained for the PF sequence (with a Peptide Ranker score of 0.99). A similar procedure applied by Mudgil et al. [49], i.e., Pepsite2 screening, following the Peptide Ranker score estimation (>0.8), led to identifying 18 peptides in camel milk hydrolysates that were considered to be DPP-IV inhibitors.

According to the theoretical predictions, all 24 sequences were non-allergenic, non-toxic, poorly water-soluble, and the majority of them were hydrophobic. Protein hydrolysates and small peptides are generally non-toxic and less allergenic than completely or partially hydrolyzed proteins [50]. The hydrophobicity value of peptides is an important factor during their separation with reversed-phase liquid chromatography. The hydrophobic character of peptides also determines the effectiveness of their action as DPP-IV and ACE inhibitors, as well as antioxidants [51]. Thus, despite their certain limitations, deploying databases of biological information (like, e.g., BIOPEP-UWM) to search for the biological functions of peptides seems to be a promising solution to this issue [8].

Phenylalanine (Phe), tryptophan (Trp), proline (Pro), leucine (Leu), and glycine (Gly) were dominant in the amino acid composition of the 24 peptide sequences selected for further LC-Q-TOF-MS/MS identification. It is well-known that the specific amino acid composition of peptides determines their bioactivity [52]. Hsu et al. [53] reported that the presence of hydrophobic amino acids, i.e., Pro, Leu, Phe, Val, Ala, and Gly, in the peptide sequence is important for DPP-IV inhibition, with Pro being the most important one. It is also noteworthy that all five peptides with the lowest *p*-value, i.e., PF, PW, PGL, PL, and PR, contained proline. For example, peptides containing proline which possesses a unique cyclic structure, can introduce kinks into the ACE structure and strongly interact with other amino acid residues [13]. The presence of proline-rich peptides has been shown to contribute to the stability of the wheat gluten hydrolysate exposed to the action of digestive enzymes [28]. Apart from Pro, other amino acids, such as Phe, Arg, His, Tyr, and Trp, have also been proven to be important for the enzymes [54]. In turn, Trp, Tyr, Pro, Ala, and Phe were also found as the amino acids most frequently present in peptides with antioxidative activity [55]. For example, Roblet et al. [56] observed that, after ultrafiltration, a fraction of the pepsin–pancreatin hydrolysate of soybean protein (<10 kDa) exhibited a higher antioxidative activity compared to the fraction with a higher molecular weight or to the whole hydrolysate. They found high contents of Trp, Tyr, and Phe in the most active hydrolysate fraction [56]. According to Sarmadi and Ismail [57], Phe, Trp, Tyr, and His are able to transform radicals into stable molecules by donating electrons.

In the next part of our study, we were able to separate in vitro digests of oat kernel proteins with liquid chromatography coupled with mass spectrometry by applying the data from in silico research. Based on the adopted criteria that were discussed earlier, 24 sequences of oat-derived peptides, identified by the bioinformatics data processing, were selected for further in vitro identification. Those sequences which were identified in the samples of oat kernel digests were assigned to the category described as ‘yes’ (see Table 2). The selected sequences were also screened for the ‘multifunctional’ biological properties using the BIOPEP-UWM database. Citing the words by Iwaniak and Mogut [8]: ”there is no a universal peptide sequence that would show all bioactivities”. This statement referred to cardiometabolic syndrome-preventive peptides occurring in cheeses and might be applicable to the results presented herein. Among the peptides selected for identification using mass spectrometry, the VW and MY (see Table 2) peptides revealed triple activities, i.e., DPP-IV-inhibitory, ACE- inhibitory, and DPPH free radical scavenging one, whereas 12 peptides: MF, GF, GW, IW, GL, PW, SF, TF, PL, TW, PR, and VF, revealed two activities, i.e., DPP-IV combined with ACE inhibitory activity or antioxidant properties. Nine mono-functional peptide sequences were also recognized, including MW, PF, HF, HW, NF, SW, ML, and MR ones, being DPP-IV inhibitors, as well as IF and PGL being ACE inhibitors.

Thirteen sequences, out of twenty-four presented in Table 2, were identified based on the analysis of their fragmentation ions (i.e., daughter ions) with the mass-to-charge ratio computed using bioinformatic tools [58]. Their identification involved the search for daughter ions typical of the selected precursor ions. The following peptide sequences were identified: GF, HF, HW, IF, ML, PL, PR, PW, TF, TW, VF, VW, and SF.

The molecular formula, retention time, mass of an ionized peptide (*m*/*z*), and mass of fragmentation ions formed after peptide fragmentation were calculated for each of the 13 identified peptides (see Table S3). The *m*/*z* values obtained for the fragmentation ions were compared with analogous values available in the METLIN database [59]. 

**Figure 3:**
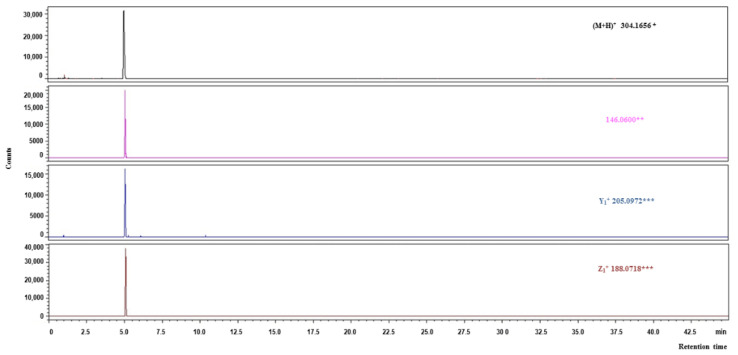
RP-HPLC-MS/MS chromatogram of the multifunctional peptide VW (DPP-IV and ACE inhibitory activity and antioxidant activity), including ion mass/charge ratios, identified in oat kernels proteins digest; * *m*/*z* of precursor ion, ** *m*/*z* of the ion present in the spectrum from the METLIN database, *** type of ion and *m*/*z* of the daughter ion.

**Figure 4:**
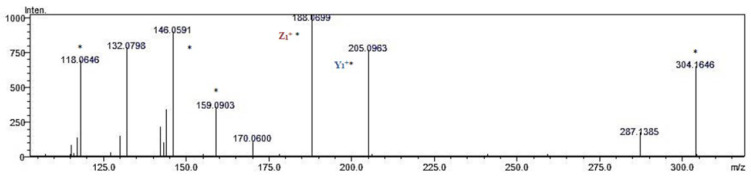
MS/MS spectrum of multifunctional peptide VW identified in oat kernels proteins digest (* mass of fragmentation ions also appearing on METLIN spectra). Fragment ion nomenclature according to Roepstorff and Fohlman (1984) [60].

An example of a chromatogram of fragment ions obtained from the VW triple active peptide (DPP-IV inhibitor/ACE inhibitor/antioxidative) is shown in Figure 3. According to the results of the in silico analysis, this sequence was released from other than globulins oat proteins fraction. The retention time of the precursor ion with *m*/*z* was 304.1656 Da, and that of the corresponding fragment ions was 5.15 min. An example of the MS/MS spectrum of the VW peptide from the analyzed samples is provided in Figure 4. The *m*/*z* ratios of z and y fragment ions allowing peptide identification were consistent with these predicted theoretically, with the precision achievable by the mass spectrometer applied. Analysis of the MS/MS spectrum enabled the finding of fragment ions y1+, z1+, and a2+ from the VW peptide. The spectrum contained also fragment ions that are not attributed to the known peptide fragmentation products [58] but are present in the reference spectrum annotated in the METLIN database.

The LC-MS method has been successfully employed by various research groups to identify peptide inhibitors of DPP-IV and ACE enzymes and antioxidative peptides. The use of bioinformatic tools available in the BIOPEP-UWM database enabled the detection of the PW fragment in a pepsin–trypsin–chymotrypsin hydrolysate of carp proteins [25]. Next, the presence of this peptide was confirmed with the LC-MS/MS method in the in vitro hydrolysate produced with pepsin and Corolase PP. In turn, Garcia-Vaquero et al. [47] used a combination of in silico and in vitro methods in their study concerning ACE peptide inhibitors derived from *Ulva lactuce* macroalgae hydrolysate. After in vitro hydrolysis with papain, 48 peptides showing ACE inhibitory activity were identified with the LC-MS/MS method. Afterward, simulated digestion in the human gastrointestinal tract was performed using enzymes from the BIOPEP-UWM database. Subsequently, a total of 86 peptides were selected, 48 of which were explicitly identified with the LC-MS/MS method. In turn, Taga et al. [61] harnessed this method to identify peptide inhibitors of DPP-IV in wheat gluten hydrolysate obtained with ginger protease. 

Bioactive peptides and proteins can be identified using standard proteomic or peptidomic techniques that include mass spectrometry coupled with the in silico methods and employing one of the two strategies: ‘top-down mimicking’ and ‘bottom-up mimicking’ [62]. The first mentioned strategy was used in the present study and, in brief, relies on supporting the experiment with the application of in silico studies. Results of the in silico identification of peptides in oat kernel protein sequences were verified using liquid chromatography with mass spectrometry. The coupling of in vitro and in silico methods to investigate peptides is also called the integrated approach [63]. In our experiment, the combined assessment was also based on the so-called ‘positive selection’, meaning that peptide search in the oat protein digests was dependent on the matching of the sequences that were experimentally recognized as peptide DPP-IV and/or ACE inhibitors and/or antioxidants [63].

The results of in vitro and in silico identification of peptides may differ, which is a common fact in the literature [8]. The possible factors affecting such differences were discussed by Iwaniak et al. [63]. Shortly, they can be divided into theoretical and experimental ones. The first includes: the number of peptides with known specific activities that can be found in the database when using software (i.e., database) and software options ‘considering’ only the specificity of enzyme when simulating the hydrolysis of protein. The latter include: the nature of protein–enzyme interactions, kinetic conditions of an enzymatic reaction, and possible modifications of protein (substrate) and peptide (product), which are not registered by the database due to its limitations concerning the annotation of sequences using specific codes [63]. Finally, according to Bucholska and Minkiewicz et al. [64], there is no perfect method that would enable identifying all possible peptides in a hydrolysate sample. 

### 2.7. Transepithelial Transport of Peptides

In our study, monolayers of Caco-2 cells were used as a model of absorption in the human small intestine. The intestinal epithelial barrier is the first gateway to actively interact with proteins after they are digested. Caco-2 cell lines have been commonly used as a tool for the duodenal digestion stage in absorption studies due to their functional and morphological similarities believed to be an equivalent of enterocytes in the small intestine in vivo [65]. The Caco-2 cells demonstrate proteome expression similar to jejunal enterocyte and brush border membrane peptidases in a similar way to the human intestinal epithelium, which, jointly with the tight junction at the apical side and carrier-mediated transport systems as well as microvillus structure, makes them a viable model for bioavailability studies [66]. The absorption of intact peptide sequences through the Caco-2 cell line, as a preclinical model for predicting intestinal permeability, has been demonstrated for multifunctional peptides released from lupine storage proteins by gastrointestinal enzymes [67], corn gluten-derived peptides [68], and dairy proteins [65]. Ample studies on the transport of bioactive peptides across enterocytes have reported their different mechanisms. It has been assumed that the passive pathways via the paracellular route or transcytosis are most often in the carrying of biopeptides, with the peptide transporters occurring less frequently [65,69].

Thirteen peptides identified in the oat kernel digest after human-like gastrointestinal digestion were selected for the experiment concerning apical to basolateral transport appointed peptide. The following aspects have been taken into account during this selection: (i) a relatively high value of PeptideRanker score combined with (ii) at least two types of biological activity demonstrated, and (iii) a relatively low Pepsite2 *p*-value. The following four peptides emerged as suitable for Caco-2 analysis: PW (DPP-IV inhibitor and antioxidative peptide), TF (DPP-IV and ACE inhibitor), VF (DPP-IV and ACE inhibitor), and VW (DPP-IV and ACE inhibitor as well as an antioxidative peptide). All selected peptides were characterized by a Peptide Ranker score higher than 0.8. 

**Figure 5:**
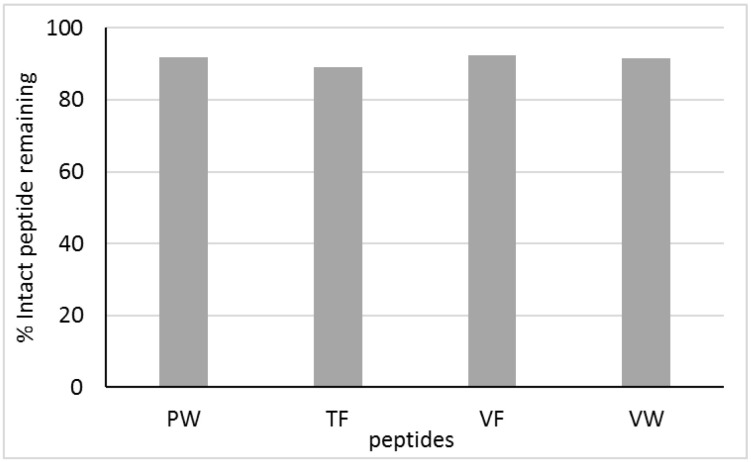
The percentage of peptides remaining intact after 2 h Caco-2 cell absorption. Each bar represents the mean and standard deviation of three replicates.

The PW, TF, VF, and VW peptides were detected on the basolateral side (Figures S1–S4). Although all the peptides studied were degraded to a certain degree by peptidases present on the Caco-2 cell surface, the extent of this degradation was small. All four peptides analyzed were rather resistant to proteolysis with Caco-2 peptidases. Among them, TF was the most susceptible to enzymatic hydrolysis, with 89% of the original peptide added to the apical chamber not having been broken down after 2 h in the basolateral compartment (see Figure 5). For the other peptides, the extent of degradation ranged from 7.5% to 8.5%. Rohm et al. [70] noticed that the almost complete disappearance of peptides from the apical compartment in the cell line could indicate their low resistance to hydrolysis. Consistently, the TF, VW, VF, and TW peptides were relatively resistant to proteolysis by peptidases present on the Caco-2 cell surface. Udenigwe and Aluko [71] found that small peptides, mostly composed of two or three amino acid residues, could exceed the intestinal absorption capacity and stability against proteases compared to polypeptides. In turn, Lacroix et al. [72] applied Caco-2 cell monolayers to study the stability and transport of five milk-derived peptides, namely LKPTPEGDL, LPYPY, IPIQY, IPI, and WR. All five peptides were susceptible to the action of brush border membrane peptidases. Nevertheless, between 70% and 92% of the peptides remained intact after 2 h of Caco-2 cell absorption. The WR and LKPTPEGDL peptides were the most resistant to Caco-2 peptidases.

Although the ability of bioactive peptides to penetrate membranes is limited, certain enzyme-inhibiting peptides or antioxidative peptides exhibit high activity in vivo. In the present study, the oat-derived peptides selected for further in vitro identification and cell line studies were composed mostly of two amino acids. It has been proved that dipeptides and tripeptides can cross the intestinal epithelium, reach the systemic circulation intact, and effectively react with respective receptors [73]. Moreover, some studies have proven the thesis that the enzyme-inhibiting peptides and antioxidative peptides derived from food proteins could first serve as endogenous inhibitors in the duodenum and then in the blood plasma [74].

## 3. Materials and Methods

### 3.1. Chemicals and Reagents

Peptides PW (>99.0%), TF (>97.6.0%), VW (>98.9%), and VF (>99.0%) were synthesized by Novazym Polska s.c. (Poznań, Polska). Pepsin from porcine gastric mucosa (EC 3.4.23.1, P7012), ACE from rabbit lung (EC 3.4.15.1, A6778), recombinant human DPP-IV (EC 3.4.14.5, D3446), α-glucosidase from *Saccharomyces cerevisiae* (EC 3.2.1.20, G0660), porcine bile extract, hippuryl-histidyl-leucine (HHL), 1,1-diphenyl-2-picrylhydrazyl (DPPH), (±)-6-hydroxy-2,5,7,8-tetramethylchromane-2-carboxylic acid (Trolox), Gly-Pro *p*-nitroanilide hydrochloride, *p*-nitrophenyl α-D-glucopyranoside (*p*NPG), glutathione, Folin–Ciocalteu’s phenol reagent, pyridine, benzenesulfonyl chloride (BSC), 1% non-essential amino acids, 5% antibiotic–antimycotic solution and phosphate-buffered saline (PBS), trypsin, EDTA, diprotin, acarbose, and captopril were purchased from Sigma-Aldrich (St. Louis, MO, USA), and an LMW-SDS Calibration Kit was purchased from Cytiva (Marlborough, MA, USA). Corolase^®^ PP was obtained from AB Enzymes (Darmstadt, Germany). Bovine serum albumin (BSA) was purchased from Bio-Rad (Hercules, CA, USA). Caco-2 cell line was obtained from the European Collection of Authenticated Cell Cultures (ECACC, England). Dulbecco’s modified eagle medium (DMEM) and fetal bovine serum (FBS) were purchased from GibcoChemical Co. (Grand Island, NY, USA). Other reagents and chemicals were of analytical grade and commercially available. LC-grade water (18 MΩ cm) was prepared using a Milli-Q PLUS (Millipore Corp., Burlington, MA, USA). 

### 3.2. Materials

The study was conducted with kernels of common oats (*Avena sativa* L.) of Krezus variety, harvested in 2019 at an agricultural farm Jeruty (53°32′ N 21°10′ E) in north-eastern Poland. 

### 3.3. In Silico Digestion

#### 3.3.1. Dataset Construction

A total of 256 amino acid sequences of oat kernel proteins (*Avena sativa* L.) available in the UniProt database (http://www.uniprot.org/uniprot; accessed on 10 December 2019) [75] were tentatively selected for in silico analysis. The next step of the proteins’ selection involved their categorization into two groups: globulins and other oat proteins. The UniProt database comprised even several dozen amino acid sequences of certain proteins sharing the same names and functions. Identical sequences were eliminated using the ClustalW2-Multiple Sequence Alignment program with default settings (http://www.ebi.ac.uk/Tools/msa/clustalw2/; accessed on 12 December 2019) [76]. Finally, a total of 115 amino acid sequences of oat kernel proteins with less than 90% identity were selected for further analyses (see Table S1). 

#### 3.3.2. In Silico Evaluation of Bioactive Peptides Potential

The proteolysis simulation tool, available in the BIOPEP-UWM database [77], was employed to predict the release of peptide inhibitors crucial in CMS pathogenesis as well as antioxidative peptides. The gastric digestion stage was simulated with pepsin [EC 3.4.23.1], whereas the duodenal stage with sequentially acting pepsin, trypsin [EC 3.4.21.4], and chymotrypsin [EC 3.4.21.1]. The hydrophobicity of peptides was calculated according to Kyte and Doolitle scale [78] as the sum of hydrophobicity values of individual amino acids present in a peptide sequence. An online peptide calculator PepCalc (http://pepcalc.com/; accessed on 5 March 2020), was applied to predict the solubility of released peptides. Their toxicity was scored in ToxinPred using an ‘SVM (Swiss-Prot) based’ method and an SVM threshold of 0.0 (http://www.imtech.res.in/raghava/toxinpred/multi_submit.php; accessed on 12 March 2020) [79]. In turn, AllerTOP (http://www.ddg-pharmfac.net/AllerTOP/; accessed on 18 April 2020) [80] was applied to analyze the immunogenic potential of peptides. The bioactivity of in-silico-released peptides was estimated with tools available on a Peptide Ranker server (http://distilldeep.ucd.ie/PeptideRanker/; accessed on 26 May 2020) [48]. The PeptideRanker tool can order a set of peptides and—based on the function-structure model—assign a score for a given peptide in the range from 0 to 1. The highest score denotes the most active peptides, whereas the lowest one indicates the least active peptides. Pepsite2 (http://pepsite2.ruselllab.org; accessed on 3 June 2020) was used to compute the potential interaction (*p*-value) between the peptide and enzymes [81]. All peptides were analyzed against DPP-IV (PDB ID: 2ONC) and ACE (PDB ID: 1O8A), which sequences were selected from the RCSB Protein Data Bank (https://www.rcsb.org/acc/; accessed on 20 June 2020) [82]. The peptides for which the PeptideRanker Score was more than 0.75 and the *p*-value was 0.01 or less were selected for identification with the LC-Q-TOF-MS/MS method.

### 3.4. Extraction of Oat Kernel Proteins

Proteins were extracted from oat kernels according to the method described by Ma [83] with slight modifications. Dry and dehulled oat kernels were ground in an electric mill. Their weighted portions were mixed with a 1 M solution of NaCl for 30 min using a mechanical stirrer. The resulting suspension was centrifuged at 21 °C and 5000× *g* for 45 min (Sigma 3K30 laboratory centrifuge, SIGMA Laborzentrifugen GmbH, Germany). The supernatant was immediately collected, whereas the precipitate was poured with the same volume of the NaCl solution, and the above procedure was repeated twice. The supernatants were combined and dialyzed (30 kDa MWCO) at 4 °C for 24 h with distilled water and then lyophilized (Feezone 4.5 lyophilizer, LABCONCO, Kansas City, MO, USA). The extraction process was repeated 3 times, and the extracts obtained were combined.

### 3.5. In Vitro Human-Like Gastrointestinal Digestion Using the INFOGEST Static Model 

The in vitro digestion, simulating digestive processes in the human gastrointestinal tract, was performed with the INFOGEST in vitro harmonized static model [84] coming from the INFOGEST cost action. Lyophilized oat kernel protein extracts (5 mg) were dissolved in 1 mL of water, and three types of samples were prepared, namely: 0, G, D. They referred to intact, i.e., undigested, gastric, and duodenal phase-digested samples, respectively. In samples G and D, pepsin was used in a dose corresponding to 2000 U/mL of digestive fluid. In turn, a Corolase PP preparation was used in sample D in a dose corresponding to 100 U/mL of a digestive fluid, whereas bile salt extract in a dose of 4000 U/mL of a digestive fluid. After each stage of digestion, the samples were stirred and incubated at 37 °C (Memmert 100–800 laboratory incubator, Germany) using a KL-942 rocking platform shaker (JWElectronic, Warszawa, Poland). The gastric and duodenal digestion lasted 120 min each. The pH value of the samples was 3.0 and 7.0, respectively. The resulting digests were lyophilized and stored at −40 °C for further analyses. 

### 3.6. Protein/Peptide Content

Protein/peptide content of the samples of all parts of the study was determined with the method of Lowry et al. [85], based on a standard curve plotted for bovine serum albumin in a concentration range from 0.1 to 1 mg/mL.

### 3.7. Sodium Dodecyl Sulfate Polyacrylamide Gel Electrophoresis (SDS-PAGE)

Sodium dodecyl sulfate polyacrylamide gel electrophoresis (SDS-PAGE) method, according to Laemmli [86], using a ‘Mini-PROTEAN’ electrophoresis kit (Bio-Rad, USA), was used to monitor the digestion process. Analyses were conducted with a 12% separating gel, a 4% stacking gel, and an LMW-SDS Marker Kit (GE Healthcare Life Sciences, Chicago, IL, USA). The gels obtained were scanned (ImageScaner III, GE Healthcare, Great Britain), and the resulting images were analyzed.

### 3.8. Enzyme Inhibition Assays

DPP-IV inhibitory activity was evaluated using a spectrophotometric method [87]. In brief, 0.025 mL of 1.6 mM Gly-Pro *p*-nitroanilide hydrochloride substrate was added to 0.025 mL of the digest dissolved in 100 mM TRIS-HCl buffer (pH 8.0). The samples were ultrafiltrated (centrifugation at 10,000× *g*, 30 min; 3 kDa MWCO). The whole mixture was stirred and incubated at 37 °C for 10 min. The reaction was initiated by adding 0.050 mL of a DPP-IV solution (0.4 mU) and continued at 37 °C for 60 min. Afterward, it was stopped by adding 0.1 mL of 1 M acetate buffer (pH 4.0). Diprotin A was used as a positive control in this assay. Absorbance was measured at a wavelength of 405 nm (GENESYS 6 spectrophotometer, Thermo Fisher Scientific, Waltham, MA, USA). The inhibition [%] of dipeptidyl peptidase IV was calculated using the following formula [88]:

DPP-IV inhibition [%] = (A_1_ − A_2_)/(A_1_ − A_3_) × 100 (1)

where: A_1_—absorbance of the sample containing TRIS-HCl instead of the potential inhibitor; A_2_—absorbance of the sample containing the potential inhibitor (oat protein digest); A_3_—absorbance of the sample containing TRIS-HCl instead of the enzyme solution and the potential inhibitor.

α-Glucosidase inhibitory activity was evaluated using the method provided by Ahmed et al. [89]. Samples were ultrafiltrated (centrifugation at 10,000× *g*; 30 min; 3 kDa MWCO). Next, 0.1 mL of an α-glucosidase solution (10 U/mL, in 0.1 M phosphate buffer, pH 6.8) was added to 0.1 mL of a sample solution (in phosphate buffer, pH 6.8). The mixture was stirred and incubated at 37 °C for 10 min. The reaction was initiated by adding 0.05 mL of *p*-nitrophenyl-α-D-glucopyranoside, then the sample was incubated at 37 °C for 20 min, and the reaction was finally stopped by adding 2 mL of a 0.1 M solution of Na_2_CO_3_. Acarbose was used as a positive control in this assay. The amount of released *p*-nitrophenol was measured at a wavelength of 410 nm using the following formula:

α-glucosidase inhibition [%] = (A_1_ − A_2_)/A_1_ × 100 (2)

where: A_1_—absorbance of the sample containing buffer instead of the potential inhibitor; A_2_—absorbance of the sample containing the potential inhibitor (oat proteins digest).

ACE inhibitory activity was evaluated using the spectrophotometric method by Jimsheena and Gowda [90]. The samples were ultrafiltrated (centrifugation at 10,000× *g*; 30 min; 3 kDa MWCO). The inhibitory activity was determined at 37 °C in a mixture composed of 0.125 mL of 0.05 M borate buffer (pH 8.2) containing 0.3 M NaCl, 0.05 mL of a 5 mM solution of HHL, and 0.025 mL of an ACE solution (5.0 mU) preincubated for 15 min with an aqueous solution of the sample (1:1, *v*/*v*). The reaction was stopped after 30 min by adding 0.2 mL of a 1 M HCl solution. Then, 0.4 mL of pyridine and 0.3 mL of BSC were sequentially added to the samples, which were carefully mixed by turning upside-down for ca. 1 min, and cooled on ice. Captopril was used as a positive control in this assay. Absorbance was measured at a wavelength of 410 nm. The inhibition [%] of angiotensin-converting enzyme was calculated using the following formula [91]:

ACE inhibition [%] = (A_1_ − A_2_)/(A_1_ − A_3_) × 100 (3)

where: A_1_—absorbance of the sample containing water instead of the potential inhibitor; A_2_—absorbance of the sample containing the potential inhibitor (oat proteins digest); A_3_—absorbance of the sample containing water instead of the enzyme solution and the potential inhibitor. 

The percent inhibition curves were plotted from a minimum of five determinations for each sample concentration and mean IC_50_ values using Graph Pad Prism^®^ v. 5.02 for Windows (GraphPad Software, San Diego, CA, USA; accessed on 21 June 2021). The IC_50_ values were defined as the inhibitor concentration ensuring the DPP-IV/α-glucosidase/ACE inhibition rate of 50%. 

### 3.9. DPPH^•^ Scavenging Activity Assay

Analyses were conducted with the method provided by Borawska et al. [25]. Samples (0.5 mL) were mixed with the same volume of DPPH (0.15 mM) in 95% ethanol. After 30 min, absorbance was measured at 517 nm. The scavenging effect was calculated using the following formula:

DPPH [%] = (A_2_ − A_1_)/A_2_ × 100 (4)

where: A_1_—absorbance of the tested sample, A_2_ –absorbance of the control sample. 

The results were calculated as the amount of Trolox µmol equivalent to 1 g of hydrolysate activity. 

### 3.10. Identification of Peptides Using Reversed-Phase Liquid Chromatography Coupled with Mass Spectrometry with Quadrupole Time of Flight Analyzer (LC-Q-TOF-MS/MS)

The samples were dissolved in a 0.1% (*v*/*v*) solution of formic acid in water to the concentration of 2.0 mg/mL lyophilizate. Next, 500 μL of the resulting solutions were collected in a test tube with a filter (Amicon^®^ Ultra Centrifugal Filters, Merck KGaA, Darmstadt, Germany; 3 kDa MWCO) and centrifuged at 21 °C and 14,000× *g* for 20 min. The LCMS-9030 assembly (Shimadzu, Tokyo, Japan) was used. The separation was performed on a bioZen™ Peptide XB-C18 column (Phenomenex, Torrance, CA USA) having the following parameters: column size—150 × 2.1 mm; particle diameter—2.6 µm; and pore diameter—100 Å; using a 0.1% (*v*/*v*) solution of formic acid in water (solution A) and in acetonitrile (solution B). The separation was performed in a gradient of 5–50% B within 30 min. Afterward, the column was flushed and equilibrated using the following gradient: 50 to 100% B from 30 to 31 min, 100% B from 31 to 36 min, 100 to 5% B from 36 to 37 min, and 0% B from 37 to 45 min. The separation was carried out at a temperature of 40 °C, injection volume of 5 μL, and flow rate of 0.4 mL/min. During separations, the collision energy (CE) reached 25, and spread CE ranged from 10 to 40. The flow rates of the spraying and drying gas were at 3.0 and 10.0 L/min, respectively. In turn, the flow rate and temperature of the heating gas were at 10.0 L/min and 300 °C, respectively. Data were collected for 30 min with a frequency of 2 kHz. Results of analyses were stored and processed using LabSolutions Ver. 5.95 software (Shimadzu, Kyoto, Japan).

Chromatograms were processed with the Savitzky and Golay method using 11 neighboring points for smoothing [92]. The range of the analyzed mass-to-charge ratios was from 30 to 2000 Da. Peptides were identified according to the procedure proposed by Darewicz et al. [39]. The mass-to-charge ratio (*m*/*z*) for the sought peptides and their fragmentation (daughter) ions was calculated using a Fragment Ion Calculator (http://db.systemsbiology.net:8080/proteomicsToolkit/FragIonServlet.html; accessed on 8 June 2021). Identification of peptides was additionally enabled by the use of data from the METLIN database [59] with the MS Search 2.3 software of National Institute of Standards and Technology, Gaithesburg, USA.

### 3.11. Caco-2 Transportation Experiments

#### 3.11.1. Caco-2 Cells Culture

Cells were cultured in Dulbecco’s modified Engle’s medium (DMEM, Sigma-Aldrich) supplemented with a 10% fetal bovine serum (FBS, Gibco), 1% non-essential amino acids (NEAA, Sigma-Aldrich), and a 5% antibiotic–antimycotic solution (Sigma-Aldrich). Incubation was carried out at 37 °C in a humidified atmosphere of 5% CO_2_ in air, and the medium was changed every 2–3 days. After the cells had reached a confluence of 80–90%, trypsinization was performed using 0.1% trypsin and 0.02% EDTA in PBS (Sigma-Aldrich). Caco-2 cells were counted with handheld automated cell counter (Scepter™ 2.0 Cell Counter, Merck Millipore) using appropriate sensors (60 µm Scepter Cell Counter Sensors, Merck Millipore). The Caco-2 cells used in this study were in 45–48 passages. For the transport experiment, Caco-2 cells were seeded in cell culture inserts with polycarbonate membranes (parameters: 30 mm diameter, 0.4 μm pore size, 4.2 cm^2^ grown surface area, Millicell Cell Culture Insert, Millipore) at a cell density of 0.5 × 10^6^/insert, and incubated in six-well culture plates. The medium was changed every second day in the volumes of: 1.5 mL on the apical side (AP) and 2.0 mL on the basolateral side (BL), as previously described by Jarmołowska et al. [93].

#### 3.11.2. Transport Studies

The confluent monolayers were used for the experiment 21 days after seeding to study the cellular transport of the synthetic peptides (PW, VF, VW, and TF). The integrity of the cell monolayers was determined by the measurement of transepithelial electrical resistance (TEER) with voltohmmeter (Millicell ERS-2 Voltohmmeter, Millipore). Only cell monolayers with TEER values higher than 300 Ω × cm^2^ were used for transport studies [94]. Inserts with the Caco-2 cell monolayers were rinsed twice with Hank’s balanced salt solutions (HBSS, Sigma-Aldrich) and preincubated with 1 mM Diprotin A (IPI, Sigma-Aldrich) for 30 min before starting the experiment. Afterward, the tested peptides (at a concentration of 1 mg/mL HBSS) were added to the apical side of inserts. After 2 h of incubation, the absorbed permeates on basolateral side were collected, frozen at −80 °C, and freeze-dried.

### 3.12. Statistical Analysis 

All experiments were repeated at least three times, and all data are presented as means ± standard deviation. Statistical analyses were carried out using Statistica v.10 (StatSoft Polska, Cracow, Poland) and applying ANOVA Kruskal–Wallis test. Differences between mean values were found statistically significant at a *p* ≤ 0.05.

## 4. Conclusions

Digests and peptides of oat kernel proteins, exhibiting multiple biological functions and important for CMS prevention, were obtained after applying the INFOGEST static model of human-like gastrointestinal digestion. This study is the first one that delivers new evidence for the multifunctional bioactive properties of peptides released from oat proteins, which could be harnessed in cardiometabolic syndrome prevention. It concerns the ability of oat digests and released peptides to modulate the activity of CMS key enzymes (i.e., DPP-IV, ACE, and α-glucosidase) and antioxidant power using in vitro models as well as the cellular intestinal membrane level. The experimental model integrating in vitro and in silico methods with cell-based assays enabled identifying peptides present in the oat kernel protein digests along with those that were able to cross the intestinal barrier. The multiple activities of digests and peptides released from oat kernel proteins suggest they could be deployed in the prophylaxis of cardiometabolic syndrome symptoms, e.g., obesity, increased postprandial glycemia, or hypertension. Although natural peptide inhibitors of DPP-IV, α-glucosidase, and ACE, as well as antioxidative peptides derived from oat kernels, are less effective than synthetic drugs, they could be used to complement pharmacotherapy in CMS management. Despite some limitations, our methodology becomes the common strategy that can be harnessed to study proteins and their hydrolysates as sources of biopeptides, including those that may act as CMS-preventive ones. To the best of our knowledge, oat protein digests were not analyzed as the reservoir of CMS-preventive peptides using the integrated in silico and in vitro methods with the INFOGEST protocol and supplemented by methods using cell lines. Our integrated in vitro and in silico approach combined with the cell-based assay led to identifying 13 out of 24 peptides. Four of them were resistant to peptidases secreted by Caco-2 cells. The results obtained seem to be promising; therefore, further in vivo studies involving, e.g., animal models are required to obtain more insights into oats as an anti-CMS diet-supportive food.

## Figures and Tables

**Table 1:** Bioactivity of digested oat kernel proteins expressed as half maximal inhibitory concentration (IC_50_), where 0—intact sample, G—sample after 2 h gastric phase, D—sample after 2 h duodenal phase (see Section 3). Value means ± SD of three determinations. The same superscripts (^a, b, c^) in the same column indicate significant differences between samples (*p* < 0.05).

Sample	DPP-IV Inhibitory Activity	α-Glucosidase Inhibitory Activity	ACE Inhibitory Activity	DPPH Scavenging
	IC_50_ (mg/mL)			μM Trolox/ mg Sample
0	10.82 ± 0.04 ^a^	25.20 ± 0.06 ^ab^	34.52 ± 0.11 ^ab^	7.93 ± 0.07 ^a^
G	4.23 ± 0.05 ^a^	10.35 ± 0.03	8.89 ± 0.07	9.91 ± 0.15 ^b^
D	0.51 ± 0.07 ^a^	1.55 ± 0.04 ^a^	0.82 ± 0.10 ^a^	33.93 ± 0.26 ^abc^
Positive control	0.20 ± 0.04 ^a^ (Diprotin A)	0.21 ± 0.17 ^b^ (Acarbose)	0.20 ± 0.08 ^b^ (Captopril)	290.00 ± 0.18 ^abc^ (Gluthatione)

**Table 2:** The predicted amino acid sequences and properties of dipeptidylpeptidase (DPP-IV), angiotensin converting enzyme (ACE)-inhibiting, and antioxidative (AO) peptides matching the oat kernel protein sequences that were selected for the LC-MS/MS identification in the oat kernel digests (see Materials and Methods).

No.	Amino Acid Sequence	Precursor Protein	Activity	PeptideRanker Score	Pepsite2 *p*-Value	Identication	BIOPEP-UWM ID	Kyte-Doolittle Hydrophobicity	Water Solubility	Toxicity	Potential Allergencity
1	MF	Other proteins	DPP-IV	1.00	1.876 × 10−3	No	3385	4.7	poor	No	No
			ACE		7.218 × 10−4		8827				
2	MW	Other proteins	DPP-IV	1.00	1.876 × 10−3	No	8690	1	poor	No	No
3	GF	Globulins, other proteins	DPP-IV	0.99	6.047 × 10−4	Yes	8782	2.4	poor	No	No
			ACE		4.579 × 10−4		7591				
4	GW	Other proteins	DPP-IV	0.99	1.934 × 10−3	No	8787	−1.3	poor	No	No
			ACE		7.708 × 10−4		7579				
4	PF	Globulins, other proteins	DPP-IV	0.99	1.512 × 10−5	No	9505	1.2	poor	No	No
5	PW	Other proteins	DPP-IV	0.99	4.843 × 10−5	Yes	8865	−2.5	poor	No	No
			AO		-		8190				
6	HF	Globulins, other proteins	DPP-IV	0.95	3.062 × 10−3	Yes	8791	−0.4	poor	No	No
8	HW	Other proteins	DPP-IV	0.95	5.719 × 10−3	Yes	8798	−4.1	poor	No	No
9	IF	Other proteins	ACE	0.95	2.086 × 10−3	Yes	7593	7.3	poor	No	No
10	SF	Globulins, other proteins	DPP-IV	0.95	1.348 × 10−2	Yes	8891	2	poor	No	No
			ACE		1.621 × 10−3		7685				
11	IW	Other proteins	DPP-IV	0.94	1.155 × 10−2	No	8807	3.6	poor	No	No
			ACE		2.086 × 10−3		7544				
12	NF	Globulins, other proteins	DPP-IV	0.94	9.678 × 10−4	No	8842	−0.7	poor	No	No
13	SW	Globulins, other proteins	DPP-IV	0.93	2.446 × 10−2	No	8896	−1.7	poor	No	No
14	ML	Globulins, other proteins	DPP-IV	0.89	4.143 × 10−3	Yes	8832	5.7	poor	No	No
15	PGL	Globulins, other proteins	ACE	0.86	7.404 × 10−5	No	7507	1.8	poor	No	No
16	MR	Other proteins	DPP-IV	0.85	4.676 × 10−3	No	8836	−2.6	poor	No	No
17	MY	Other proteins	DPP-IV	0.84	1.126 × 10−2	No	8838	0.6	poor	No	No
			ACE		5.894 × 10−3		3388				
			AO		-		8090				
18	TF	Globulins, other proteins	DPP-IV	0.83	8.881 × 10−4	Yes	8900	2.1	poor	No	No
			ACE		1.143 × 10−3		8185				
19	VF	Globulins, other proteins	DPP-IV	0.82	2.740 × 10−3	Yes	8917	7	poor	No	No
			ACE		3.016 × 10−3		3384				
20	GL	Globulins, other proteins	DPP-IV	0.81	3.881 × 10−3	No	8561	3.4	poor	No	No
			ACE		3.881 × 10−3		7599				
21	PL	Globulins, other proteins	DPP-IV	0.81	4.843 × 10−5	Yes	8638	2.2	poor	No	No
			ACE		2.612 × 10−5		7513				
22	TW	Other proteins	DPP-IV	0.81	2.839 × 10−3	Yes	8913	−1.6	poor	No	No
			AO		-						
23	VW	Other proteins	DPP-IV	0.82	5.018 × 10−3	Yes	8928	7	poor	No	No
			ACE		2.034 × 10−3		3486				
			AO		-		8461				
24	PR	Globulins, other proteins	DPP-IV	0.80	2.964 × 10−5	Yes	9489	−6.1	poor	No	No
			ACE		6.661 × 10−6		3537				

## Data Availability

The data presented in this study are available on request from the corresponding author. The data are not publicly available due to this study is an industrial work and some results are confidential.

## Supplementary Materials

The following supporting information can be downloaded at: https://www.mdpi.com/article/10.3390/molecules27227907/s1, Figure S1: MS/MS spectra of fragment ions obtained from the precursor ion with *m*/*z* = 302.15 from the “gastrointestinal” hydrolysate after in vitro digestion of oat kernel proteins (after Caco-2 experiment) obtained during the identification of the PW sequence peptide using the Q-TOF mass spectrometer: * mass of fragmentation ions appearing also on METLIN spectra [60]; Figure S2: MS/MS spectra of fragment ions obtained from the precursor ion with *m*/*z* = 267.13 from the “gastrointestinal” hydrolysate after in vitro digestion of oat kernel proteins (after Caco-2 experiment) obtained during the identification of the TF sequence peptide using the Q-TOF mass spectrometer: * mass of fragmentation ions appearing also on METLIN spectra; Figure S3: MS/MS spectra of fragment ions obtained from the precursor ion with *m*/*z* = 265.15 from the “gastrointestinal” hydrolysate after in vitro digestion of oat kernel proteins (after Caco-2 experiment) obtained during the identification of the VF sequence peptide using the Q-TOF mass spectrometer: * mass of fragmentation ions appearing also on METLIN spectra; Figure S4: MS/MS spectra of fragment ions obtained from the precursor ion with *m*/*z* = 304.17 from the “gastrointestinal” hydrolysate after in vitro digestion of oat kernel proteins (after Caco-2 experiment) obtained during the identification of the VW sequence peptide using the Q-TOF mass spectrometer: * mass of fragmentation ions appearing also on METLIN spectra; Table S1: Amino acid sequences of oat kernel proteins (*Avena sativa* L.) selected for in silico studies; Table S2: Peptides with antioxidant activity and cardiometabolic syndrome (CMS) key enzymes inhibition activity encrypted in oat protein sequences and released using in silico digestion and their overall solubility [77]; Table S3: Peptides identification in oat kernel digests.

## Sample Availability

Samples of the oat hydrolysates are available from the authors.
