# Supplementary material for: Multi-Bioactivity of Protein Digests and Peptides from Oat (Avena sativa L.) Kernels in the Prevention of the Cardiometabolic Syndrome
Source: Molecules. 2022 Nov 15;27(22):7907. doi: 10.3390/molecules27227907 (PMC9695537; doi:10.3390/molecules27227907)
Supplement: Supplementary file 1 [file molecules-27-07907-s001.zip › molecules-1982115-supplementary.pdf]

Table S1. Amino acid sequences of oat kernel proteins (*Avena sativa* L.) selected for *in silico* studies.

No	UniProt ID	Name of protein	Amino acid sequence	Amino acid chain lenght	Mass (kDa)
			**Globulins**		
1	Q38779	11S globulin	MATTSFPSMLFYFCIFLLFHGSMAQLFGQSSTPWQSSRQGGLRGCRFDRLQAFEPLRQVRSQAGITEYFDEQNEQFRC<br>TGVSVIRRVIEPQGLVLPQYHNAPALVYILQGRGFTGLTFPGCPATFQQQFQPFDQSQFAQGQSQSQTIKDEHQRVQR<br>FKQGDVVALPAGIVHWCYNDGDAPIVAIYVFDVNNNANQLEPRQKKFLLAGNNKFLLAGNNANQLEPRQKEFLL<br>AGNNKREQQSGNNIFSGLSVQLLSEALGISQQAAQGSKSNDQRGRVIRVSQGLQFLKPIVSQQVPVEQQVYQPIQTQ<br>DVQATQYQVGQSTQYQVGKSTPYQGGQSSQYQAGQSWDQSFNGLEENFCSLEARKNIENPQHADTYNPRAGRITR<br>LNSKNFPILNIVQMSATRVNLYQNAILSPFWNINAHSVIYMIQGHARVQVVNNNGQTVFSDILHRGQLLIVPQHFVV<br>LKNAEREGCQYISFKTNPNSMVSHIAGKTSILRALPIDVLANAYRISRQEARNLKNNRGEEFGAFTPKLTQTGFQSYQ<br>DIEEASSSAVRASE	551	61.862
2	O49258	12S globulin	MATTSFPSVLFYSCIFLLYNGSMAQLFGQSFTPWQSSRQGGLKGCKFDRLQAFEPLRQVRSQAGVTEYFDEQNEQFRC<br>TGVFVIRRVIEPQGLLLPQYHNAPGLVYILQGRGYTGLTFPGCPATFQQQFQPFDQAQDQSQSHLKDEHQRVHRFKQ<br>GDVIALPAGIVHWGYNDGDAPVVAIYVFDVNNNANQLEPRQKEFLLAGNNKEDQQFGQNIFSGFNIQLLSEALGIS<br>QQAAQRIQSQKEQRGEIIRVTQALQFLKPTMSQQELVEHQAYQPIQSQEGQSTQYQVGQSTQYQEGQSTQYQAGQS<br>QDRSFNGLEENFCSLEARQNIGNPKRADTHNPRAGRITRLHGQNFPILNLVQMSATRVNLYQNAILSPFWNINAHSV<br>VYMIQGHAQVQVVNNNGQTVFNDRLRQGQLLIVPQHYVVLKKAEREGCQYISFKTNPNSMVSHIAGKSSILRALPV<br>DVLANAYRISRQEARNLKNNRGQESGVFTPKFTQTSFQPYPEGEDESSLTNKASE	515	58.225
3	P12615	12s seed storage globulin 1	MATTRFPSLLFYSCIFLLCNGSMAQLFGQSFTPWQSSRQGGLRGCKFDRLQAFEPLRQVRSQAGITEYFDEQNEQFRC<br>AGVSVIRRVIEPQGLLLPQYHNAPGLVYILQGRGFTGLTFPGCPATFQQQFQQFDQARFAQGQSKSQNLKDEHQRVH<br>HIKQGDVVALPAGIVHWCYNDGDAPIVAVYVFDVNNNANQLEPRQKEFLLAGNNKREQQFGQNIFSGFSVQLLSE<br>ALGISQQAAQKIQSQNDQRGEIIRVSQGLQFLKPFVSQQGPVEHQAYQPIQSQQEQSTQYQVGQSPQYQEGQSTQYQ<br>SGQSWDQSFNGLEENFCSLEARQNIENPKRADTYNPRAGRITHLNSKNFPTLNLVQMSATRVNLYQNAILSPYWNIN<br>AHSVMHMIQGRARVQVVNNHGQTVFNDILRRGQLLIIPQHYVVLKKAEREGCQYISFKTTPNSMVSYIAGKTSILRA<br>LPVDVLANAYRISRQESQNLKNNRGEEFGAFTPKFAQTGSQSYQDEGESSSTEKASE	518	58.545
4	Q38794	A.sativa seed storage protein	MKNFLILALLSMAATMATAQFDPSEQYQPYPEQQQPILQQQLLLQQQQMLLQQQPLLQVLQQQLNPCRQFLVQQ<br>CSPVAAVSFLRSQILQQSSCQVMRQQCCRRLEQIPEQLRCPAIHSVVQAIIMQQQQQFFQPQMQQQFFQPQMQQV<br>TQGIFQPQMQQVTQGIFQTQMQGQIEGMRAFALQALPAMCDVYVPPHCPVATTPLSGF	209	24.066
			**Other oat proteins**		
5	P27919	Avenin	MKIFFFLALLALVVSATFAQYAESDGSYEEVEGSHDRCQQHQMKLDSCREYVAERCTTMRDFPITWPWKWWKGGC<br>EELRNECCQLLGQMPSECRCDAIWRSIQRELGGFFGTQQGLIGKRLKIAKSLPTQSTWALSAISPNSMVSHIAGKSSILR<br>ALPVDVLANAYRISRQEARNLKNNRGQESGVFTPKFTQTSFQPYPEGEDESSLINKASE	214	24.23

6	I4EP86	Avenin	MKTFLILVVLAMAATMATAQYDPSEQYQPYPEQQPFMQQQQPFMQQQQQPFMQQQQPFMQQQQQPFMQPLLQQQMIPCQMFLMQQCNPVEMVPFLRSQILRQSTCHVMRKQCCRQLAQVPKQLRCPAIHSMVHAIVMQQQQVVQAQQMGLVQPQTQMAGQVFIQPQQLPQYQAMKVVAMQTLPAMCNVQVPPYCSTGQVSGIAAGIGGC	209	27.839
7	I4EP88	Avenin	MKNFLILALLAMAATMATAQFYPSEQYQPYPEQQQPFLQQQPLLQQQQLLQVLQQQLNPCRQFLVQQCSPVAEVPFLRSQILQQSSCQVMKQQCCRQLAQIPEQVRCPAIHSVVQAIILQKLQQQLLQPQLQQQLLQPQLQQQILQAQLQQQLLQAQVQQQLLQPQVQQQLQQQLIQPQLQQVFIPPQLQQVFQPQLQQVFQPQQQAQFEGMRAFALQALPAMCDVYVPPHCSVATTPLG VV	242	27.839
8	Q09072	Avenin	MKTFLIIALLAMAVATATATTVQYNPSEQYQPYPEQQEPFVQQQQPFVQQQQPFVQQQQMFLQPLLQQQLNPCKQFLVQQCSPVAAVPFLRSQILRQAICQVTRQQCCRQLAQIPEQLRCPAIHSVVQSIILQQQQQQQQFIQPQLQQQVFQPQLQLQQQVFQPQLQQQVFQPQLQQVFNQPQMQGQIEGMRAFALQALPAMCDVYVPPQCPVATAPLGGF	222	25.471
9	Q2EPY2	Avenin	MKTFLIFALLAMAATMATAQFDPSEQYQPYPEQQQPILQQQQMLLQQQQQQMLLQQQPLLQVLQQQLNPCRQFLVQQCSPVAVVPFLRSQILQQSSCQVMRQQCCRQLEQIPEQLRCPAMHSVVQAIIMQQQQFFQPQMQQVTQGIFQPQMQQVTQGIFQPQLQQVTQGIFQPQMQGQIEGMRAFALQALPAMCDVYVPPHCPVATAPLGGF	212	24.258
10	F2Q9W5	Avenin (Fr)	IALLAMAVATTTATTTVQYDPSEQYQPYPEQQEPFVQQQPPFVQQEQPFVQQQQPFVQQQPFVQQQPFVQQQQPFVQQQQMFLQPLLQQQLNPCKQFLVQQCSPVAAVPFLRSQILRQAICQVTRQQCCRQLAQIPEQLRCPAIHSVVQAIILQQQQQQQQFIQPQLQQQVFQPQLQLQQQVFQPQLQLQQQVFQPQLQLQQQVFQPQLQLQQQVFQPQLQQQVFQPQLQQVFNQPQQQAQFEGMRAFALQALPAMCDVYVPPHCPVATVP	271	24.632
11	F4MJY0	Avenin (Fr)	LALLSMAATMATAQFDPSEQYQPYPEQQQPILQQQLLLQQQQQMLLQQQPLLQVLQQQLNPCRQFLVQQCSPVAAVSFLRSQILQQSSCQVMRQQCCRRLEQIPEQLRCPAIHSVVQAIIMQQQQQQFFQPQMQQQFFQPQMQQVTQGIFQPQMQQVTQGIFQTQMQGQIEGMRAFALQALPAMCDVYVPPHCPVATT	198	22.817
12	F4MJY5	Avenin (Fr)	LVVLAMAATMATAQYDPSEQYQPYPEQQPFMQQQQPFMQQQQQPFMQQQQPFMQQQQQPFMQPLLQQQMIPCQMFLMQQCNPVEMVPFLRSQILRQSTCHVMRKQCCRQLAQVPKQLRCPAIHSMVHAIVMQQQQVVQAQQMGLVQPQTQMAGQVFIQPQQLPQYQAMKVVAMQTLPAMCNVQVPPYCSTGQVSGIAA	198	22.772
13	F4MJY2	Avenin (Fr)	LTLLAMAATMATAQFDPSEQYQPYPEQQQPFLQQQPLLQQQQQLLQVLQQQLNPCRQFLVQQCSPVAEVPFLRSQILQQSSCQVMKQQCCQQLAQIPEQVRCPAIHSVVQAIILQKLQQQLLQPQLQQQLLQPQLQQQLLQAQLQQQLLQAQLQQQLLQAQLQQQLLQPQVQQQLQQQLIQPQLQQVFIPPQLQQVFQPQQQAQFEGMRAFALQALPAMCDVYVP	212	26.008
14	F4MJY6	Avenin (Fr)	LLAVLAMAATMATAQYDPSEQYQPYPEQQPFMQQQQPFMQQQQQPFMQQQQPFMQQQQPFMQPLLQQQMIPCQMFLMQQCNPVEMVPFLRSQILRQSTCHVMRKQCCRQLAQVPKQLRCPAIHIMAHAIIMQQQQQQQQVVQAQQMGLVQPQTQMASQVFIQPQQLPQYQAMKVVAMQTLPAMCNVQVPPYCSTGQVGGIAA	202	23.253
15	P80356	Avenin -3	MKTFLIFALLAMAATMATAQFDPSEQYQPYPEQQQPILQQQQMLLQQQQQMLLQQQPLLQVLQQQLNPCRQFLVQQCSPVAVVPFLRSQILQQSSCQVMRQQCCRQLEQIPEQLRCPAIHSVVQAIIMQQQQFFQPQMQQQFFQPQMQQVTQGIFQPQMQQVTQGIFQPQLQQVTQGIFQPQMQGQIEGMRAFALQALPAMCDVYVPPHCPVATAPLGGF	220	25.275
16	Q09114	Avenin-E	TTTVQYNPSEQYQPYPEQQEPFVQQQPFVQQQQQPFVQQQQMFLQPLLQQQLNPCKQFLVQQCSPVAVVPFLRSQILRQAICQVARQQCCRQLAQIPEQLRCPAIHSVVQAIILQQQQQQQFFQPQLQQQVFQPQLQQVFNQPQQQAQFEGMRAFALQALPAMCDVYVPPQCPVATAPLGGF	182	21.036
17	L0L837	Gliadin	MKTFLILALLAMAATMATAQYDPSEQYQPYPEQQPFMQQQQPFMQPLLQQQMIPCQMFLMQQCSPVEMVPFLRSQILRQSTCHVMRRQCCRQLAQIPRQLRCPAIHSMVHAIIMQQQQQQQLVQAQQMGLVQPQTQMAGQVFIQPQQLSQYQTMKVVAMQTLPAMCNVQVPPYCSTGQVG GMAAGIGGC	189	21.422

18	L0L4J1	Gliadin	MKNFLIIALLAMAAAMATAQFDPSEQYQPYPEQQQPILQQQLLLQQQQMLLQQQPLLQVLQQQLNPCRQFLVQQ CSPVAAVSFLRSQILQQSSCQVMRQQCCRRLEQIPEQLRCPAIHSVVQAIIMQQQQQFFQPQMQQQFFQPQMQQV TQGIFQPQMQQVTQGIFQTQMQGQIEGMRAFALQALPAMCDVYVPPHCPVATTPLGGF	209	23.99
19	L0L5G8	Gliadin	MKNFLILALLAMAAATATATTTVQYDPSEQYQPYPEQQEPFVQQQPFVQQQQPFVQQQMFLQPLLQQQLNQCK QFLVQQCSPVAVVPFLRSQILRQAICQVARQQCCRQLAQIPEQLRCPAIHSVVQAIILQQQQQQFFQPQLQQQVFQ QQVFQPQLQQEVFQPQLQQQVFQPQLQQVFNQPQQQAQFEGMRAFALQALPAMCDVYVPPHCPVATVPLSGF	223	25.751
20	L0L4I8	Gliadin	MKTFLIFALLAMAATMATAQFDPSEQYQPYPEQQQPILQQQQMLLQQQQMLLQQQPLLQVLQQQLNPCRQFLV QQCSPVAVVPFLRSQILRQAICQVTRQQCCRQLAQIPEQLRCPAIHSVVQAIILQQQQQQQFIQPQLQQQVFQPQLQ LQQQVFQPQLQLQQQVFQPQLQLQQQVSQPQLQLQQQVFQPQLQQQVFQPQLQQVFNQPQQQAQFEGMRAFAL QALPAMCDVYVPPHCPVATVPLSGF	250	29.056
21	Q42518	(1-3,1-4)-beta-glucanase	MASQGVASMFALALLLGAFASIPQSVESIGVCYGMSANNLPAASTVVGMFKSNGINSMRLYAPDQAALQAVGGTG VNVVVGAPNDVLSALAASPAAAASWVRSNIQAYPKVSFRYVCVGNEVAGGATQNLLPAMQNVQGALASAGLGHI KVTTSVSQAILGVYSPPSAGSFTGEADAFMGPVVQFLARTGSPLMANIYPYLAWAYNPSAMDMSYALFTASGTVVQD GAYGYQNLFDTTVDAFYTAMGKHGGAGVKLVVSESGWPSAGGEAATPANARIYNQYLINHVGRGTPRHPGGIETY VFAMFNENQKDNGVEQNWGLFYPNMQHVYPISF	334	34.757
22	Q9ZPN 1	Acetyl-CoA carboxylase	QNSKDLQKVVDIVLSHQGVRSKTKLILTLMEKLVYPNPAAYRDQLIRFSSLNHKRYYKLALKASELLEQTKLSELRTSI VRNLSALEMFTEERAGFSLQEKKLAINESMGDLVSAPLPVEDALISLFDCIDQTLQQRVIETYISRLYQPQLVKDSIQLK YQDSAVIALWEFNEGHPDNRLGAMVILKSLESVSTAIGAALKDRSHYASSAGNTVHIVLLDADTQTNTTEDSGDND QAQDRMDKLSVILKQDIVTADLCAAGVKVISCIVQRDGALMPMRRTFLLSEEKLCYEEEPILRHVEPPLSALLELDKL KVKGYNEMKYTPSRDRQWHIYTLRNTENPKMLHRVFFRTLVRQPSAGNRFMSGHINDVEVGHAEESLSFTSSSILRSL MTAIEELELHAIRTGHSHMYLCILKEQKLLDLVPVSGNTVVDVGQDEATACSLMKEMALKIHELVGARMHHLSVCQ WEVKLKLDSDGPASGSWRVVTTNVTSHTCTVDIYREVEDTDSQKLVYHYAASSPGPLHGVTLSNSYQPLSVIDLKRCS ARNNRTTYCYDFPLAFETAVRKSWANVSSENNQCYVKATELVFADKNGSWGTPIVPMQRAAGLNDIGMVAWILD MSTPEFPSGRQIIVIANDITFRAGSFGPREDAFFEAVTNLACERKLPLIYMAANSGARIGIADEVKSCFRVEWVDPANP ERGFKYIYLNEEDYGRISSSVIAHKTQLDSGEIRWVIDSVVGKEDGLGVENIHGSAAIASAYSRAYEETFTLTFVSGRTV GIGAYLARLGIRCIQRIDQPIILTGFSALNKLLGREVYSSHMQLGGPKIMATNGVVHLTVPDDLEGVSNILRWLSYVPA NIGGPLPITKSLDPIDRPVAYIPENTCDPRAAISGIDDSQGKWLGGMFDKDSFVETFEGWAKTVVTGRAKLGGIPVGVI AVETQTMMQLVPADPGQPDSHERSVPRAGQVWFPDSATKTAHAMLDFNREGLPLFILANWRGFSGGQRDLFEGI	1010	112.157
23	D1L6E8	AGL6-like MADS box transcription factor (Fr)	SKLKAKFEALQRTQRHLLGEDLGPLSVKELQQLEKQLECSLSQAGQRKTQLMMEQVEELRRKERQLGEINRQLKHKL DAEGGSSNNYRAMQQITWAAGTVVDESAAAYHMHQHQNQNQNQ PNHSGAMDCEPTPA	134	15.23
24	Q071L4	Aluminum –acti-vated malate transporter (Fr)	HGQFRFRHPWSQYQKLGTLCRQCASSMEALASYVITTTKTQYPAAASPDLSSKVRKTCGEMSSHSAKVLRQLATATQ TMTVPPPANLAVSAAMTAAECLRSELAENAAILQVMHVAVTASLLADLVAQVKEIAECVDVLARHADFKNPEDAR RDVAVDSQGRSGPLPDVVIL	172	18.476
25	Q0KIX4	Anthranilate synthase	MESLAASAFSPSRLAARPSRAAAAAAVPARARAVASAGGRRRRSGKGSGAVRCCAGSSASASAVINGSAAAKAEQE DRQRFFEAAARGSGKGNLVPLWECIVSDHLTPVLAYRCLVGEDDMDTPSFLFESVEQGLEGTTNVGRYSMVGAHPV MEIVAKEDKVTTMDHEKGTVTEQIVDDPMEVPRSIMEGWHPQQIDDLPETFSGGWVGFFSYDTVRYVEKKKIPFSGA PQDDRNLPDVHLGLYDDVLVFDSVEKKVYVIHWVSLDRHASTEEAYQYGRSRLRRFLSKVHNTNVPKLSPGFVKLH TKQFGTALTKSTMTSDEYKSAVLQAKEHILAGNIFQIVLSQRFERRTYATPFEVYRALRIVNPSPYMAYVQARGCILVA	610	66.993

			SSPEILTKVQKGKIINRPLAGTTRRGKTENEDKLQEEELLSDKKQRAEHIMLVDLGRNDVGRVSKPGSVKVEKLMNIE<br>RYSHVMHISSTVSGELDDNLQSWDALRAALPVGTVSGAPKVKAMELIDQLEVTRRGPYSGGLGGISFNGDMMIALA<br>LRTIVFSTAPSHNTMFSYKNSDRRREWVAHLQAGAGIVADSIPEDEQKECENKAAALARAID LAESAFVDKE		
26	Q0KIX3	Anthranilate synthase alpha 2 subunit	MESLAASAFSPSRLAAHPSRAAAAAAVPVRARAVAAAGARRRRSGKGSSGVRCCAGSASASANAVINGSAAAKAE<br>QEDRQRFFEAAARGSGKGNLVPLWECIVSDHLTPVLAYRCLVGEDDMDTPSFLFESVEQGLEGTTNVGRYSMVGAH<br>PVMEIVAKEDKVTTMDHEKGTVTEQIVDDPMEVPRSIMEGWHPQQIDDLPETFSGGWVGFFSYDTVRYVEKKKIPFS<br>GAPQDDRNLPDVHLGLYDDVLVFDSVEKKVYVIHWVSLDRHASTEEAYKYGRSRLRRFLSKVHNTNVPKLSPGFVK<br>LHTKQFGTALTKSTMTSDEYKNAVLQAKEHILAGNIFQIVLSQRFERRTYATPFEVYRALRIVNPSPYMAYVQARGCIL<br>VASSPEILTKVQKGKIINRPLAGTTRRGKTENEDKLQEEELLSDKKQRAEHIMLVDLGRNDVGRVSKPGSVKVEKLM<br>NIERYSHVMHISSTVSGELDDDLQSWDALRAALPVGTVSGAPKVKAMELIDQLEVTRRGPYSGGLGGISFNGDMMIA<br>LALRTIVFSTAPSHNTMFSYKNSDRRREWVAHLQAGAGIVADSIPEDEQKECENKAAALARAIDLAESAFVDKE	611	67.142
27	P22220	Arginine decarboxylase (EC 4.1.1.19)	MAKNYGDVYHVEGWGEPYFAVNKDGHLCVRIYGRETLPGQEIDVLSVIEQATSADGTGKKLQFPMILRFPDVLRHRI<br>NSLHTAFANAIKYTQYGSVYQGVFPVKVNQHKDVVQDMVHFGYDHSYGLEAGSKPELLIAMSCLTKAKPGAYLVC<br>NGYKDSAYVALALAARAMGLNVIIVLEMEEELDIVIEESSKLGVEPVIGVRAKLLTKIPGHFGSTAGKHGKFGLPAEKI<br>YEVAKKLKALNKLHWLKLLHFHVGSMIPTTDIVFKAASEASDIYCALVKEYGVETMTTLDCGGGLGVDYDGTRSGSS<br>DMSVAYGLEEYASSIVQAVRLKCDYHGVPHPVLCTESGRAMASYHSMIILEALSAIPEPKDDEDEATTEQLHGRIRDL<br>SSKLQPTGLSMSSHAVHIKKHGIEMYKLGKKLSKSVTTDAHTIYNYHMNLSVFSLMPDYWGIQHLFPMMPVSRLDEK<br>PTHKATLVDVTCDSDGKVDKFIRDTETMPLHPLDPKLGGYYVAVLLTGAYQEALSNKHNLFGGPSLVRVVGTGNGG<br>AFNVEAALLGSTTEELIGTVSYDVKQDISSVIEERARENKVWEMVEKLVESGLHTMPYLAD YKPPPMA	607	66.706
28	A9L9T2	ATP synthase subunit beta	QIIGPVLDVTFPPGKLPYIYNAXVVQSRDTSDKQINVTCEVQQLLGNNRVRAVAMSATDGLMRGMEVIDTGAPLSVP<br>VGGATLGRIFNVLGEPVDNLGPVDSSATFPIHRSAPAFIELDTKLSIFETGIKVVDLLAPYRRGGKIGLFGGAGVGKTVL<br>IMELINNIAKAHGGVSVFGGVGERTREGNDLYMEMKESGVINEKNIEESKVALVYGQMNEPPGARMRVGLTALTM<br>AEYFRDVNKQDVLLFIDNIFRFVQAGSEVSALLGRMPSAVGYQPTLSTEMGSLQERIASTKKGSITSIQAVYVPADDLT<br>DPAPATTFAHLDATTVLSRGLASKGIYPAVDPLDSTSTMLQPRIVGNEHYETAQRVKETLQRYKELQDIIAILGLDELS<br>EEDRLTVARARKIERFLSQPFFVAEVFTGSPGKYVGLAETIRGFQLILSGELDGLPEQAFYLVGNIDEASTKAITLEEENK<br>SKK	474	51.285
29	Q5I7F1	Auxin-responsive protein (Fr)	YRVSIGLPGNGGWCSGWGRGDDGGAANARASVQGRGETWVRRRSGRQGCGGGGGRREEEGAGPAAKAQVVGW<br>PPIRSYRKNTMATSLSAPRSKDEVEAKQAPVAGCLYVKVSMDGAPYLRKVDLKMYKNYKDLSLELEKKFSCFTVGHG<br>ESNGKSGRDGLSDCRLMDPKSGAELVLTYED KDG	110	12.081
30	O64413	Auxin-binding protein	MESRAGIAAAVRGLRFAGAGRRGTLLLALLFVAADAFLPVAEPSCPRDNSVVKDINQMHQSNYGLEGLSHITVGGA<br>LAHGMKEVEVLLETVSAGQRTPIHRHSCEEVFVVLKGRGTLFLGSTSLKYPGTPQEIPFSQNSTFTVPINDPHQVWNS<br>DEHEDLQFLVIISRPPVKVFLYDDWSMPHTAAKLKFPFLWDEDCLAAPKDEL	206	22.648
31	Q5I7F3	Auxin-responsive protein (Fr)	YRVSIGLPGNGGWCSGWGRGDDGGAANARASVQGRGETWVRRRSGRQGCGGGGGRREEEGAGPAAKAQVVGW<br>PPIRSYRKNTMATSLSAPRSKDEVEAKQAPVAGCLYVKVSMDGAPYLRKVDLKMYKNYKDLSLELEKKFSCFTVGHG<br>ESNGKSGRDGLSDCRLMDPKSGAELVLTYEDKDG	183	19.58
32	Q38786	Avenacosidase 1	MALLCSALSNSTHPSFRSHIGANSENLWHLSADPAQKSKRRCNLTLSSRAARISSALESAKQVKPWQVPKRDWFPPE<br>FMFGAASAAYQIEGAWNEGGKGPSSWDNFCHSHPDRIMDKSNADVAANSYYMYKEDVRMLKEIGMDSYRFSISWP<br>RILPKGTLDGGINHEGIQYYNDLLDCLIENGIKPYITLFHWDTPQALADEYKDFLDRRIVKDYTDYATVCFEHFGDKV<br>KNWFTFNEPHSFCGLGYGTGLHAPGARCSAGMTCVIPEEDALRNPYIVGHNLLLAHAETVDVYNKFYKGDDGQIG	574	65.041

			MVLDVMAYEPYGNNFLDQQAQERAIDFHIGWFLEPMVRGDYPFSMRSLVGDRLPFFTKSEQEKLVSSYDFVGINYYT SRFAKHIDISPEFIPKINTDDVYSNPEVNDSNGIPIGPDVGMYFIYSYPKGLKNILLRMKEKYGNPPIYITENGTADMDG WGNPPMTDPLDDPLRIEYLQQHMTAIKEAIDLGRRTLRGHFTWSLIDNFEWSLGYLSRFGIVYIDRNDGCKRIMKKSA KWLKEFNGATKKLNNKILGASSCCSGVTHGGGTA		
33	Q9ZP27	Avenacosidase 2	MALLCSALSNSTHPSFRSHIAGANSENLWHLSAHPAQKSKRRCNLTLSSRAAARISSALESGKLKPWQIPKRDWFPPE FTFGAASAAYQIEGAWNEGGKGPSSWDNFCHNYPERIMDGSNWDVAANSYYMYKEDVRMLKEIGMDSYRFSISWP RILPEGTLEGGINHEGIQYYNDLLDCLIENGIKPYITLFHWDTPQALADKYNDFLDRRIVKDYTDYATVCFEHFGDKV KNWITFNEPHSFCGLAYGTGLHAPGLCSPGMDCAIPQGDALRQPYIVGHNLLLAHAETVDVYKKFYKGDDGQIGM VMDVMAYEPYGNNFVDQQAQERSIDFHIGWFLEPMVRGDYPFSMRSLVGDRLPFFTKSEQEKLVSSYDFVGINYYTA RFSEHIDISPEIIPKLNTDDAYSTPEFNDSNGIPIGPDLGMYWILSYPKGLKDILLLMKEKYGNPPIYITENGTADMDGW GNPPMTDPLDDPLRIEYLQQHMTAIKEAIDLGADVRGHFTWSLIDNFEWSMGYLSRFGIVYIDRNDGFKRIMKKSAK WLKEFNGATKEVNNKILGASSCCSGELMWFLVQNPYGK	578	65.693
34	Q9M4E2	Avenoindoline	MKALFLIGLLALVASTAFAQYSEVVGSYDVAGGGGAQQCPVETKLNSCRNYLLDRCSTMKDFPVTWRWWKWWKG GCQELLGVCCSRLGQMPPQCRCNIIQGSIQGDLGGIFGFQRDRASK VIQEAKNLPPRCNQGPPCNIPGTIGYYW	148	16.357
35	J7FK21	Avenoindoline	MKTLFLLALLALVAGTTFAQYSEVGGWYNEVGAGGGSQQCPMERPNLSSCKDYVMERCFTMKDFPVTWPTRWWK GGCEHEVREKCCQQLSQIAPQCRCDSIRGMIQGKLGGFFGIWRGDVFKQIQRAQRLPSKCNMGADCKFPSGYYW	148	16.787
36	Q9M4E1	Avenoindoline-b	MKTLFLLALLALVASTTLAQYSEVGGWYNEVGGGGGAQQCPLERPKLSSCMDYVMERCFTMKDFPVTWPTRWWK GGCEHEVREKCCNQLSQIAPQCRCDSIRGMIQSKFGGFFGIWRGDV FKQTQRAQSLPSKCNMGADCKFPSGYYW	148	16.721
37	A5A493	Barren inflorescence2-like serine/threonine protein kinase (Fr)	NRRFPLPSARFYAAEVLLALEYLHMMGVVYRDLKPENVLIRADGHIMLTDFDLSLQSPPRFVAEPLSARSSSFVGTHE YVAPEVASGGAHGAAVDWWAYGVFLYELLHGRTPFAGATNEETLRNIVRAPLAFPLAAARDLIARLLVKDPAARV GSLRGAADLKAHPFFRGLNFALLR	177	19.499
38	Q43076	Beta-fructofuranosidase (EC 3.2.1.26) (Fr)	ERAHVPTMASTIRSTSITPMAPLGDIVWGHAVSTDLVNWIILEPAIERDSPGDINGCWTGSATILPGGQPIVIYTGGDA ENHQVQNIMLPKNRSDPYLREWTKAGNNPVLQLVGPGFNSSQFRDPTTGWIGPDGLWRMSIGAEVNGYGAALLYK SERLSKLDYSSSPTVF	170	18.423
39	Q7XXP4	Caffeoyl-CoA 3-O-methyltransferase (Fr)	TIGLPCIEKAGVAHKIDFREGPALPVLDALLEDEANHGTFDFVFVDADKDNYLNYHQRLMKLVRVGGLLGYDNTL WNGSVVLPADAPMRKYIRYYRDFVLELNKALAADDRVEICQLPVG DGITLCRRAK	130	14.608
40	D0F043	Calmodulin (Fr)	LTVMRSLGQNPTEAELQDMINEVDADGNGTIDFPEFLNLMARKMKDTDSEEELKEAFRVFDKDQNGFISAAELRHV MTNLGEKLTDEEVDEMIREADVDGDGQINYEEFVKVMMAK	116	13.21
41	Q4A1C8	CBF-like transcription factor (Fr)	MDTGPEYNLTPTSSSSSQELGMALSSPTSPTPKRPAGRTKFKETRHPVYRGVRRRGSNGRWVCEVRVPSKSGERLWLG THVTAEAAARAHDAAMLAMHGHTSAACLNFPDSAWLLNVPSNLSDLADVRRAAIEAVVEFLRLEAIKDGAAAVA VPIDGVVASAALAPSSHADNASPAATSQPSAASEVPEALGGDMFELHTSGEMGLGTYYADLAEGLLLEPPPAAASSE HGGDCGDAPDMMRRYGNGATEIRLSLAAE	258	26.916
42	Q4A1C9	CBF-like transcription factor	MDMSGSEQWSSPSSSLEHGGPAVWTTPPKRPAGRTKFKETRHPVYRGVRRRGNAGRWVCEVRVPGQRGERLWLGT YLTAESAARAHDAAMLGLLGRSASSACLNFADSAWRLTVTPGLSDLAGVRRAALAAVANFLRREAAGGAANVPA DEDTSSASADNAGGSSATSQPYVDGTFDFEMPAGMGSDIFELDMSGEMDQGTYYAGLAEGLLLEPPPQHAGACWD TGDGGADPALWSY	237	24.955

43	Q4A1D0	CBF-like transcription factor	MDMTGSEQWSSSSSSSYSVEHGGPAVWTTPPKRPAGRTKFKETRHPVYRGVRRRGNAGRWVCEVRVPGQRGERLW LGTYLTAESAARAHDAAMLGLHGRSASACLNFADSAWLLDLPSPLSDLAAVRRVALAAVVRGQCRKLVGDIAALL CRWDIRGAGRAGQRHVRAGRVRGNGLRHVLRGPRGGIAAGAAAAARRRLLGHRRRWRS	208	22.64
44	D0U5L9	Cellulose synthase-like protein (Fr)	MGIMATGRIGPLNDESWVGVELGEDGETDESGAAVDDRPVFRTEKIKAVLLYPYRVLIFVRLIAFTLFVIWRISHKNPD AMWLWVTSICGEFWFGFSWLLDQLPKLNPINRVPDLAVLRQRFDRPDGTSTLPGLDIFVTTADPFKEPILSTANSVLSI LAADYPVDRNTCYVPDDSGMLLTYEALAEASKFATLWVPFCRKHGIEPRGPESYFELKSHPYMGRAQDEFVNDRRR VRKEYDEFKARINSLDHDIRQRNDGYNAANAHREGEPRPTWMADGTQWEGTWVDASENHRKGDHAGIVKVLLN HPSHSRQYGPPASADNPLDFSGVDVRVPMLVYVSREKRPGHNHQKKAGAMNALTRAFALLSNAPFILNLDCDHYI NNSQALRSGICFMLGRDSDTVAFVQFPQRFEGVDPTDLYANHNRIFFDGSLRALDGMQGPIYVGTGCLFRRITVYAF DPPRINVGGPCFPMLGGMFAKTKYQKPGLEMTMAKAKATPVPAKGKHGFLPLPKKTYGKSDAFVDSIPRASHPSPY VAAYNTAEGIVTDEATMAEAVNVTAAAFEKKTGWGKEIGWVYDTVTEDVVTGYRMHIKGWRSRYCSIYPHAFIGT APINLTERLFQVLRWSTGSLEIFFSKNNPLFGSTYLHPLQRIAYINITTYPFTAIFLIFYTTVPALSFVTGHFIVQRPTTMFY VYLGIVLATLLIIAVLEVKWAGVTVFEWFRNGQFWMTASMSAYLQAVCQVLIKVIFQKDISFKLTSKLPAGDGKKDP YADLYVVRWTPLMIVPIIVIFVNIIGSAVAFAKVLDGEWTHWLKVAGGVFFNFWVLFHLYPFAKGILGKHGKTPVVV LVWWAFTFVITAVLYINIPHMHSPGGKHTKVAHGHHGQKFLGWP	758	100.288
45	Q9M3W5	Chlorophyll synthase, chloroplastic	MATSHPLAAAAATSSSSATFRPPLRFLSSPPSSLTLNRRRSFPVVCAADADAKETTKKPTIPDKAPAAGSSFNQLLGIKG AKQETNIWKIRLQLTKPVTWPPLVWGVLCGAAASGNFHWTVEDVTKSIVCMLMSGPCLTGYTQTINDWYDRDIDAI NEPYRPIPSGAISENEVITQIWVLLLGGLGLGALLDIWAGHDFPIIFYLALGGSLLSYIYSAPPLKLKQNGWIGNFALGA SYIGLPWWAGQALFGTLTPDIVVLTCLYSIAGLGIAIVNDFKSIEGDRTLGLQSLPVAFGMETAKWICVGAIDITQLSV AAYLLSTGKLYYALALLGLTIPQVILQFQYFLKDPVKYDVKYQASAQPFFVFGLLVTALATSH	378	40.764
46	F5B4I6	Development-related protein kinase	MEGNTRGGGHSDALKNYNLGRTLGIGTFGKVKIAEHKHTGHKVAIKILNRRQMRTMEMEEKAKREIKILRLFIHPHII RLYEVIYTPTDIFVVMEYCKYGELFDCIVEKGRLQEDEARRIFQQIISGVEYCHRNMVVHRDLKPENLLLDSKYNVKLA DFGLSNVMHDGHFLKTSCGSPNYAAPEVISGKLYAGPEVDVWSCGVILYALLCGTLPFDDDNIPNLFKKIKGGIYILPS HLSALARDLIPRMLVVDPMKRITIREIREHPWFQNRLPRYLAVPPPDTAQQAKMIDEDTLQDVVNLGYDKDHVCESL CNRLQNEATVAYYLLLDNRFRATSGYLGAEHQSMDRSYNQVASSESASSTRNYLPGSNDPHGSGLRPYYPVERKW ALGLQSRAHPREIMIEVLKALQELNVCWKKNGHYNMKCRWCPGFPQVSDMLDANHSFVDDSTIMDNGDVNGRLP VVIKFEIQLYKTRDDKYLLDMQRVTGPQLLFLDFCAAFLTNLRVL	508	58.141
47	Q9XFF1	DNA-binding protein	MAMTPPTSFPPASPSSYFNNMSTGFLDSPILLTPSLFPSPTTGSFPLEPLNWMGTAPESNDGLQLGSVKDGQQRQYSGF TFQTTAAPVPAAMPGTNTTASSFLQSSMPMAQQGHDSYTGEQQQPWSYQDAGSMDAMTTRPASFSTPYEAPDMVG NGGYNNAPVSSSGTTAGYGRVQSRRPSSDDGYNWRKYGQKQMKGSENPRSYYKCSFAGCSTKKKVEQAPDGQVTE IVYKGTHNHPKPQNPRRSSAPASSSYASPDASSDALSGTPENSSASYGDDETNGVSSALAGQFGGGGEEFADNEPDSK RWRTDSDAEGVPVGANRTVREPRVVVQTMSDIDILDDGYRWRKYGQKVVKGNPNPRSYYKCTTAGCPVRKHVERA SQDLRAVVTTYEGKHNHDVPALRGSAAAAARYRAAPMQAASYLQGGGGGYSSLRPDGFGGGAPAQPADQSGFAL SGFDYNNSSYSYSGMQQQNDAMYYDAARTKDEPRDDMFFEQSLMF	501	53.817
48	B3VKW5	Dof-type zinc finger protein (Fr)	CPRCKSGNTKFCYYNNYSMSQPRYFCKACRLYWTHGGSLRNVPIGGGCRKPKRPGTSDAHKLGMASSSEPTGVVPPS NCTGMNFANVLPTFMSGGFDIQSSLSLTTFGSSS	111	11.949
49	P86181	Endochitinase	VSSVISSSLFEKMLLHRGFYTYDAFIAAAKSFPAFATTGSTDVRKREVAAFLAQTSHETTGGWPTAPDGPYELGSTSDY FGRGPIQISYNYNYGAAGKAIGVDLLRNPDLVTSDNTVEFKTALWFWMTPQSPKPSSHDVITGRWSPSSTDKAAGRV PGYGVLTNIIDGGVECGKGQESHV ADRIGYYKDNLDCYNQKPFA	200	21.728

50	Q9LLD7	Fructose 1,6-bisphosphate aldolase (EC 4.1.2.13)	MASATLLKSSFLPKKSEWGVTRQAAAPKPMTVSMVVRASAYADELVKTAKTVASPGRGILAMDESNATCGKRLASIGLENTEANRQAYRTLLVTPPGLGNYISGAILFEETSTSRLLMGRRLLTSLSSRVIVPGIKVDKGLVPLVGSNDESWCQGLDGLASREAAYYQQGARFAKWRTVVSIPNGPSELAVKEAAWGLARYAAISQDNGLVPIVEPESHVDGEHGIERTFEVAQKVWAETFFYMAQNNVMFEGILLKPSMVTPGAECKDRATPEEVASYTLKLLHRRIPPSVPGIMFLSGGQSVVEATLNLNAMNQAPNPWHVSFSYARALQNTCFKTWGGRPENVAAAQEALLLRAKANSLAQLGKYTSDGEAAAAKEGMFVKNYSY	388	41.922
51	Q0PLN5	Fruitful-like MADS-box transcription factor (Fr)	ISVLCDAEVAVFVFSPEGKLYEYATDSSMDKILERYECYSYAEKALISAESESEGNWCHEYRKLKAKIETIQKCHKHLMGEDLDSLNLKEPQQLEQQLESSLKHIRSRKGHLMMESIFELQKKERSLQEENKALQKELVERQKAARQQQQVQWDHQTQTQQAHQNQPQAQTSSSSSSFMIRDHQAHAPQNICYPPVTMGGEAVAAAPGQQGQLRIGGLPPWMLSHLNA	229	26.148
52	Q0PLN7	Fruitful-like MADS-box transcription factor (Fr)	ISVLCDAEVGLIIFSTKGKLYEFATDSCMDKILERYERYSYAEKVLISTESEIQGNWCHEYRKLKAKVETIQKCQKHLMGDDLESLNLKELQQLEQQLESSLKHIRSRKSQLMHESISELQKKERSLLEENKILQKELVEKQKAHTQQAQWEQTHPQTSSSSSSFMIREAPPATNTSAGNQPAAAGERSEEAAEQPQARSGLPPWMLSPL	210	23.961
53	C8ZK43	HKT1 protein (Fr)	LFLSTSALTVTGLSTVKMEDLSSSQIVVLTLLMLIGGEVFVSLLGLILRVNHQDIQDLPSVKISAVHVELEAIDLANSVALSDESQLEEAAHAIPPKSSAEFKRSRSVKCLGYSVFGYFAVVHVVGFLLVLLYITRVPTASAPLKKKGINIVLFSMSITVASFANGGLVPTNEN	174	18.57
54	Q7XXP3	Hydroxyanthranilate hydroxycinnamoyltransferase 1	MKITVRSSTVVVPAAETPRVRLWNANPDLVVPRFHTPSVYFYRRGGEDGGDACYFDAGRMRRALAEALVPFYPMAGRLAHDEDGRVEIDCNAEGVLFVEADAPDGAVDDFGDFVPTMGLKRLIPTVDFTGGISSYPLLVVQVTHFKCGGVALGIAMQHHVADGFSGLHFINSWSDLCRGVPIAVMPFIDRTLLRARDPPVPTHPHIEYQPAPAMLGSEEPQALAGKPESPPTAVDIFKLSRSDLGRLRAQLPTGEGAPRFSTYAVLGAHVWRCASLARGLAPEQPTKLYCATDGRQRLTPTHPDGYFGNVIFTATPLAEAGKVTGSLADGATTIQDALEKMDDEYCHSALDYLELQPDLSALVRGAHTFRCPNLGLTSWVRLPIHDADFGWGRPVFMGPGGIAYEGLAFVLPSANRDGSL SVAISLQAEHMEKFRKMIFDF	441	32.574
55	Q7XXP0	Hydroxyanthranilate hydroxycinnamoyltransferase 4 (Fr)	LVLQVTYFKCGGVSLGVGMQHHVADGMSGLHFINSWSDLCRGAQIAVMPFIDRTLLRARDPPTPSYTHVEYQPAPAMLSSAPQALSGKPTLAPTAVDIFKLTRSELGRLRAQLPTGEGAPRFSTYAVLAAHVWRCVSLARGLPAEQPTKLYCATDGRHRLQPRCRRVTSETSSSPHPLAEAGKVTGAVADGAAVIQGALDRMSDDYCRSALDYLETQPDLSALVRGAHTFRCPNLGLTSWVRLPIHNADFGWGRPVFMGPGGIAYEGLAFVLPSANKDGSLSIAISLQAEHMEKFRKLIADV	302	32.574
56	B7UCU7	Iron/phytosiderophore transporter	MDVLGPDRTRIAPEIEKHVAAEGDRESDPALAAERELEPLGRWQDELTVRGMVAALLIGFIYTVIVMKIALTTGLVPTLNVSAALLSFLALRGWTRLLDRFGIVSRPFTRQENTIVQTCGVACYTIAFAGGFGSTLLGLNKNTYELAGDSPGNGPGSYKEPGIGWMTAFLFSCSFGGLLTLIPLRQVLVVDYRLVYPSGTATAVLINGFHTAQGDKNSRKQIRGFLKYFGGSFLWSFFQWFYTGGDVCGFIQFPTFGLKAWKQTFFFDFSLTYIGAGMICPHIVNISTLLGAILSYGILWPLISKNKGDWYPADVKESSMKSLYGYKAFICIALIMGDGLYHFTKIITITCKGMYRQFSRKHADNREKNVDNTVSLEDLQRDEVFKRGHLPAWIAYSGYAVLSVVAVVTTPIMFRQVKWYYVVIAYVVAPMLGFANSYGTGLTDINMGYNYGKIGLFVFAGWAGRDNGVVAGLVVGTCVKQLVLISADLMQDFKTSYLTKTSPRSMMVAQAIGTAMGCVVSPLTFMLFYRAFDIGNPDGTWKAPYALIYRNMAILGVEGFSVLPKYCLALSGGFFAFAAILSIARDFTPHRYRQYVPLPMAMAVPFLVGGSFAIDMCVGSLVVFIWNKINKKEAGFMVPAVASGLICGDGIWTFPSSILALAKITPPICMKFTPAP	676	74.234
57	Q8LT03	Leaf thionin Asthi 1	MGSIKGLKSVVICVLVLGIVLEQVQVEGKSCCKDIMARNCYNVCRIPGTPRPVCATTCRCKIISGNKCPKDYPKLHGDPDAGTPNAIEFCNTGCMSSICDNMNNAYNVEDKEIDVELCGN ACTSFCNQIIVRASVAA	137	14.684

58	Q8LT01	Leaf thionin Asthi 3	MGSIKGLRSLIMCVLIVGIVLEQVQVEGNTCCKDDIARNCYNVCRIPGTPTFICANMCRCIITRRNECPNDYPKLQSDL DAGTPNAIEFCNMGCMSSICGNMKNAYPGEEKENDKEFCSIACARFCNKITVSTSVAA	137	14.981
59	Q6PL61	Leafy hull sterile 1 (Fr)	IENKISRQVTFAKRRNGLLKKAYELSLLCDAEDALIIFSGRGRLFEFSSSSCMYKTLERYRTCNYNSQEAAPLVENEINY REYLKLKTRVEFLQSSQRNILGEDLGPLSMKELDQIESQIDVSLKHIRSRKNQVLLDQLFDLKSKEQELQDQNKDLRKK LQEMSCGENAVHMSWQDGGGQSSSSGQAVDPYPGPLQHPEHDSSMQIGYHQTYMEQLBNEDHTASHHPNAQPGS SAGWI	238	27.226
60	Q672R1	Lipase 1	MKLLPCILSILLLASVDPAISSVRRFDSIFSFGDTFADTGNGRVVYAENSVPDPTAHPPYGQTFFGHPTGRSTDGRLIIDF IAHELWLPLVPPSLSRNASFSHGASFAVSAATALDVGFFKDIPIAGMLALDTSLRVQLQWFESLKTSLCGPAKACPPGF FDKSLFFMGEFGVNDYSFSLLGKTLAQVRSIVPDVVKAIAEATEGLIHHGAKTVVVPGIPPLGCTPPNLVFFPSADPAG YEPRTGCLKGFNELSVHHNTLLQEALETVQTNNPGALVVYADFYTPVIKMVKSPWKYGLTTKVLSCCCGGGGKYNF NMSAGCGMPGASVCEDPSQYLYWDGHFTEAAHRKIARGWLRKLNMHDLMMEPAYPYFDMAKDSA	379	41.061
61	G1JSL5	Lipoxygenase	MLLGGLIDNLTGANKHARLKGTVVLMRKNVLDLNDFGATIIDGVSEFLGKGVTCQLISSTVVDNNNGNRGKVGAE AGLEQWLTSLPSLTTGESKFGLTFDWEVDKLGVPGAIVVNNYHSAQFFLKTITLDDVPGRAGKLTFVANSWIYPAEK YRYNRVFFANDTYLPSQMPAALKPYRDDELRNLRGDDQQGPYEEHDRVYRYDVYNDLGEDRPVLGGTADHPYPRR GRTGRKPNPNDPSLESRLSLLEQIYVPRDEKFGHLKMSDFLGYSIKAITQGILPAVRTYVDCTPGEFDSFQDIINLYEGGI KLPKIAALEELRKRFPFQLLKDLLPVGGDFLLKLPLPHIIKEDKQAWRTDEEFAREVLAGVNPVMITRLTEFPPKSTLDP SKYGDHTSTITAAHIEKNLEGLTVQQALEGNKLYILDHHDRFMPFLIDVNNLDGNFIYATRTLFFLRGDGRLTPLAIEL SEPFIQDGLTTAKSKVYTPVPSGSVEGWVWELAKAYVAVGDSGWHQLVSHWLNTHAVMEPFVIATNRQLSVTHPV HKLLSPHYRDTMTINALARQTLINAGGIFEMTVFPGKFALGMSSVVYKDWNFAEQGLPDDLLRRGVAVPDPSSPYKV RLLIEDYPYAADGLAIWHAIEQYVTEYLAIYYPDDAVLQDDVELQAWWKEAREVGHGDLKDAPWWPSMQTVAEL AKSCATIIWIASALHAAVNFGQYPYAGYLPNRPTVSRRRMPEPGTQEYAELERDPERAFIHTITSQIQTIIGISLLEVLSK HSSDELYLGQRDTPEWTSDPKALAVFQRFSDRLVDIESKVVGMNHDPQLKNRNGPAKLPYMLLYPNTSDRKGDAA GLTAKGIPNSISI	862	96.664
62	H6ULY 1	MADS5-like protein (Fr)	AKRRNGLLKKAYELSVLCDAEVALIIFSTRGRLFEFSTSSCMYKTLERYRSCNYNSQATATPETELSNYQEYLKLKTRVE FLQTTQRNLLGEDLGPLSMKDLEQLENQIEISLKNIRSTK SQQSLDQLFELKRKEQQLQDVNKDLRRKI	149	17.464
63	Q9MU Z6	Maturase K (Intron maturase)	MEKFEGYSEKHKSCQQYFVYPLLFQEYIYAFAHDYGLNDSEPVEIISCNNKKFSSLLVKRLITRMYQQNFWINSINHPN QDRLLDYKIGFYSEFYSQILPEGFSIVVEIPFSLRELSCPKEKEIPKFQNLRSIHSIFPFLEDKFLHLDSISHIEIPYPIHLEILV QLLQYRIQDVPSLHLLRFFLNYYSNWNSFITSMKSIFLFKKENKRLFRFLYNSYVSEYEFLLVFLRKQSSCLPLSSSGTFLE RIIFSRKMEHIGIMYPSFFRKTIWFVMDPLMHYVRYQGKAILASKGTHFLNKKWKWYLINLWQYLFSFWTQPRRVHL NQLANSCFDFLGYLSGVPKSSLLVRNQMLENLFLIDTRMKKLDTIVPVTALIGYLSKAQFCTGSGHPISKPIWTDLSDW DILDRFGRICRNLFHYHSGSSKKQTLYRLKYILRLSCARTLARKHKSTVRTFMQRLGSAFLEEFFTEQELVFSLMFTKTS LFSFRGSHSERIWYFDIIRINDLVKPLN	511	61.154
64	Q43379	Mitogen-activated protein kinase	MDGAPVAEFRPTMTHGGRFLLYNIFGNQFEITSKYQPPIMPIGRGAYGIVCSVMNFETREMVAIKKIANAFDNNMDA KRTLREIKLLRHLDHENIVGLRDVIPPSIPQSFNDVYIATELMDTDLHHIIRSNQELSEEHCQYFLYQLLRGLKYIHSAN VIHRDLKPSNLLLNANCDLKICDFGLARPSSESDMMTEYVVTRWYRAPELLLNSTDYSAAIDVWSVGCIFMELINRAP LFPGRDHMHQMRLITEVIGTPTDDDLGFIRNEDARRYMRHLPQFPRRPFPGQFPKVQPAALDLIERMLTFNPLQRITV EEALEHPYLERLHDVADEPICTDPFSFDFEQHPLTEDQMKQLIFNEALELNPNFRY	369	42.871
65	Q9XFN 2	Transcription factor	MYRVKSESDCEMMHEDQMDSPLADDGGSSGGSPHRGGGPPLKKGPWTSAEDAILVDYVKKHGEGNWNAVQKNT GLFRCGKSCRLRWANHLRPNLKKGAFTPEEERLIIQLHSKMGNKWARMAANLPGRTDNEIKNYWNTRIKRCQRAG	546	59.188

			LTIYPASIINQSANEDQQGSSDFNCGENLSNNLLNGNGLYLPDFTCDNFIANSEALSYAPQLSAASISNLLGQSFASKG<br>CGFMDQAGMLKQSDSLLPGLSDTINGALSSVDQFSNDSEKLKQALGFDYLHEANSSSRIIAPFGGALTGSHAYLNGTF<br>SASRSTNGPLKMELPSLQDTESDPNSWLKYTVAPTMQPTELVDPYLHSPAPTPSVKSECASPRNSGLLEELIHEAEALR<br>SGKNQQPSVRSSSSSVSTPCDTTVVSPEFDLCQEYWEEHPSTMLVQEYAPFSGTESTAPASAASPDVFQLSKISPAESHS<br>LGSGEQVMEPSYEPGAGDTPPHPENLRPDAFFSGNTSDSSLFNNAIAMLLGNDLNTECKPVLFDPSSWSNMPHACQ<br>MSEEFK		
66	Q9AV9<br>9	Myo-inositol-1-<br>phosphate<br>synthase	MFIESFRVESPNVRYGAGEIESEYRYDTTELVHESHNGASKWVVRPKSVNYHFKTDTNVPKLGVMLVGWGGNNGST<br>LMAGVIANREGISWATKDKVQQANYFGSLTQASTIRVGSYNGEEIYAPFKSLLPMVSPDDIVFGGWDISSMNMADA<br>MTRAKVLDIDLQKQLRPYMESIVPLPGIYDPDFIAANQGSRANNVIKGTKKEQMEQVIKDIREFKEKNKVDKVVVLW<br>TANTERYSGVSVGLNDTMENLLASVDKNEAEISPSTLYAIACVMEGVPFINGSPQNTFVPGLIDLAIKNNCLIGGDDF<br>KSGQTKMKSVLVDFLVGAGIKPTSIVSYNHLGNNDGMNLSAPQTFRSKEISKSNVVDDMVASNNILYEPGEHPDHV<br>VVIKYVPYVGDSKRAMDEYTSEIFMGGKNTIVLHNTCEDSLLAAPIILDLVLLAELSTRIQLKAEGEEKFHSFHPVATIL<br>SYLTKAPLVPPGTPVVNALAKQRAMLENIMRACVGLAPENNMILEYK	510	56.13
67	A9LA0<br>4	NADH<br>dehydrogenase<br>subunit F	IMSMGFGLFFIPTATKNLRRIWAFPSVLLLSIAMVFSVQLSIQQINGSSIYQYLWSWTVNNDFSLEFGYLIDPLTSIMLILI<br>TTVGILVLIYSDGYMSHDEGYLRFFVYISFFNTSMLGLVTSSNLIQIYFFWELVGMCSYLLIGFWFTRPIAASACQKAFVT<br>NRVGDFGLLLGILGFFWITGSLEFRDLFKIANNWIPNNGINSLLTTLCAFLLFLGAVAKSAQFPLHVWLPDAMEGPTPI<br>SALIHAATMVAAGIFLLARLLPLFISLPLIMSLISLVGTITLFLGATLALAQRDIKRSLAYSTMSQLGYMMLALGIGSYQA<br>ALFHLITHAYSKALLFLGSGSIIHSMEPLVGYSPDKSQNMVLMGGLRKYIPITRSAFLWGTLSLCGIPPLACFWSKDEIL<br>SNSWLYSPFFGIIASFTAGLTAFYMFRIYLLTFDGSLRVHFQNYSSTKEGPLYSISLWGKRIPKGVNRGFILSTTKNGVSFF<br>LQNIPKIQGNTTNRIGSFSTSFGAKKTFAYPHETENTMLFPLLILLLFTLFIGFIGISFDNGATDNGIGGLTILSKWLTPSIN<br>FTQESSNSSINSYEFFTNAIYSVSLAIFGLFIAYIFYGSSYSFFQNLDLQNSFYKESPFSYFFDKIKKNIYSWSYNRGYIDIFYT<br>RVFTFGIRALTELTEFFDKGVIDGITNGVGLVSFCIGEEIKYVGGGRISSYLFFFLCYVSVFLFFFLS	721	80.813
68	D8WQ<br>Z4	NAD(P)H-qui-<br>none oxidoreduc-<br>tase subunit H,<br>chloroplastic	MSLPLTRKDLMIVNMGPQHPSMHGVLRLIVTLDGEDVIDCEPILGYLHRGMEKIAENRTIIQYLPYVTRWDYLATMFT<br>EAITVNAPEFLENIQIPQRASYIRVIMLELSRIASHLLWLGPFMADLGAQTPFFYIFRERELIYDLFEAATGMRMMHNYF<br>RIGGVAADLPYGWIDKCLDFCDYFLRGVVEYQQLITQNPIFLERVEGVGFIRGEEAVNWGLSGPMLRASGIQWDLRK<br>VDPYESYNQFDWKVQWQKEGDSLARYLVRVSEMRESIKIIQQAVEKIPGGPYENLEVRRFKKAKNSEWNDFEYRFLG<br>KKPSPNFELSKQELYVRVEAPKGELGIYLVGDDSLFPWRWKIRPPGFINLQILPQLVKKMKLADIMTILGSIDIIMGEVD<br>R	393	45.768
69	E025R4	NBS-LRR type re-<br>sistance protein<br>(Fr)	GSMLRMGELVRLWVAQGFVKAVCATEDMDDVAERYIKELVSCSFMQPIETASGTRFRIHDVVHDLLDKVAKNCFRI<br>ENAPFKQEKFSLKKKANMNARSRREEVWGGHIPRDVQHLFVQNYDGDLITEEILGLENLRTLIIYVVQEDSPVEEKIID<br>SICKRLLELRVLAVAFSREHDPIKQANKFPVPESISQLKHLSYLAFRTSSSWTVILPKTELNHIQVLDFGDGKIFEFTSAG<br>LINLRHIFCSSIMKFPNIGRLTSLQTIPKVCVSNERGCDVKQLRNLNKLRGSLEISG	293	33.431
70	E025R6	NBS-LRR type re-<br>sistance protein<br>(Fr)	SFKMRKEDLVHLWIAQGFVKTRCATEDMEDIAEGYIQELVSCSFLQPEKSRNRELFRIHDLLHDLADMVTSTDCFIIEN<br>ARSHRGESWKGNIPQDVRHLFIQNYDAELITEKTLGLRQLLTLFINFVGEDTTVEEKVIESICKRLPKLRVLAIAFSQVY<br>YPIKKPNRFSFPESISQLKYLRYLGFRTDYSCHISLPNTLNKLQHIQVLDLGKGSLREFMFSALVNLRHISYLHWKLRDV<br>GRLSSLQTLPCYFEVSNEQGRELKQLMDLNKLRGKLDIRG	279	32.643
71	O81526	NBS-LRR type<br>disease resistance<br>protein O1 (Fr)	GKTTLLHVFNNYLDNKVHDYQVVIFIEVSNSETLNIEEIQQTISERLNLPWNEAEPIAKRAKFLVKALTRKRFVVLLDD<br>VRKKFQLEDVGIPTPDTNSQSKLILTSRYQDICFQMNAQRSLIEMQILGNDASWELFSSKLSEEASAAVELLGSQNVIR<br>DYAMAIAQSCGGLPLALNVIGTAVAGLEESEWKSAADAIATNMHNIAGVDEMFGRLKYSFDRLTPTQQQCFLYCTL	456	51.413

			FPEYGSISKDQLVEYWLAEGFLLNDREKGYQIIRSLISACLLQASGSLSSKVKMHHIIRHLGLWLVNKSDAKFLVQPGM<br>ALDNTPSAGEWKEATRISIMSNNITELSFSPKCKTVTTLLIQNNPNLNKMSYGFFRTMSSLKVLDLSHTAITSLPECDTL<br>VALEHLNLSHTHIMRLPERLWLLKELRHLDLSVTIALEDTPEQLLKVAQVESAQPLPQPLWYP		
72	O49004	NPH1-2	MASKGAGAGGGGGGGDGYDEPQRPKQQLPRDSRGSLEVFNPSSSAVEPPSAFRPAARSASPFIEEVAGGIEDVGKAT<br>QRAAEWGLVLQTNEQTGRPQGVSARSSGGGGSARSSSDDKAVAGAIPRVSEELRAALSAFQQTFVVSDASRPGHPIM<br>YASAGFFNMTGYTSKEVVGRNCRFLQGSGTDPAEIAKIRQALADGSNYCGRVLNYKKDGTAFWNLLTIAPIKDEEGR<br>VLKFIGMQVEVSKYTEGNKDTAVRPNGLPESLIKYDARQKDQARSSVSELLLAIKNPRSLSESTNSTFKRKSQESVGPLT<br>GDRPGKRSSESGSRRNSKSGARTSLQKISEVPERGNKSRKSGLYSLMSLLGMGPGNIEKDMLKPRDEDPLLDSDDERPE<br>SFDDELRRKEMRRGIDLATTLERIEKNFVITDPRLPDNPIIFASDSFLQLTEYSREEILGRNCRFLQGPETDRATVRKIRD<br>AIDNQTEVTVQLINYTKSGKKFWNLFHLQPMRDQKGDVQYFIGVQLDGTEHVRDAAEREGVMLIKKTAENIDEAA<br>KELPDANLRPEDLWANHSKVVLPKPHMKDSASWRAIQKVLEGGENIDLKHFRPVKPLGSGDTGSVHLVELLNTGEY<br>FAMKAMDKNVMLNRNKVHRANAEREILDMLDHPFLPTLYASFQTKTHICLITDYYPGGELFLLLDRQPLKVLREDA<br>VRFYAAEVVIALEYLHCQGIIYRDLKPENILLHRDGHISLTDFDLSCLTSCRPQVFLPEEANKKSRRKSRSSPVFFAEPM<br>RASNSFVGTEEYIAPEIITGAGHTSAVDWWALGILLYEMLYGYTPFRGKTRQRTFANILHKDIRFPASISVSLPARQLIY<br>RLLHRDPSNRLGSYEGSNEIKEHPFFRGINWALVRGTAPPKLDAPLFPDGTDKGLGDATAAADNHTDMF	927	102.855
73	H6ULZ<br>6	Panicle phytometr<br>2-like protein (Fr)	ELRRIENKISRQVTFAKRRNGLLKKAYELSILCDAEVALVLFSHAGRVYQFSSSSNMVKTLERYQRYIFASQDAVVPTR<br>DEIQNNYQEYMELKARVEVLQHSQRNLLGEDLAPLNTSELDQLEGQVGKSLRQIRSRKTQVLMDELCGLKRKEQML<br>QDANLTLKRKLDEMELDAAPPPTQQQQQQERPWQQNDRVPVPSQTPPQPDHFFQRLR	213	24.935
74	Q38769	Permatin	MASSSVLQLLPLLMLAITIATTDAATITVVNKCSYTVWPGALPGGGVRLDPGKSWALNMPAGTRGARVWPRTGCTF<br>DASGRGHCVTGDCGGALACRVSGQQPATLAEYTLGKGGAKDFFDLSVIDGFNVPMSFQPVGGAACRGATCAADIT<br>KQCPQELKVAGGCASACGKFGGDTYCCRGQFTDKCPPTNYSKFFKGKCPDAYSYAKDDQTSTFTCPVGTNYQIVLC<br>P	228	23.676
75	O81525	Peroxidase PXC6	MGSASCISLRVMLVALAATAASAQLSSTFYDTSCPKALATIKSGVAAAVSSDRRMGASLLRLHFHDCFGCDASVLLSG<br>NEQNAAPNAGSLRGFSVIDNIKTQVEAVCKQTVSCADILAVAARDSVVALGGPFLEQVPLGRRDSTSATGNTGDLPA<br>PTSSLAQLQAAFSKKNLDTTGMVALSGAHTIGQAQCKNFRSRIYGGDTNINAAFATSLQANCPQATGGSGDSSLAPL<br>DTKTPNAFDNSYYNNLLSQKGLLHSDQVLFNNGTTDNTVRNFASSASAFTGAFTTAMIKMGNISPLTGTQGQIRLSC<br>SKVNS	314	32.338
76	G1JSL4	Peroxygenase 1	MAEDAVVSDAVVVSDAMSSVAKGAPVTAQRPVRDDLEKHIPKPYLARALVAVDVNNPEGTKGGRHEHGQKSVLQ<br>QHVSFFDQNGDGIIYPWETFRGLRRLGFNLIVSFIVAIGIHTGLSYPTLPTWRPSLLFPVYIDRIHKAKHGSDTATFDTEG<br>RFMPVNFENIFSKNARSQPDKLTLREIWMMTNDHRLAYDPFGWVANKGEWILLYMLAKDDEGYLPKEAIRGVYDG<br>SLFEFLAEQR TKKAHGKQH	249	28.107
77	D0E576	Phenylalanine<br>ammonia-lyase	MAGNGPICEKDPLNWAAAAAEITGSHLDEVKRMVALSREPVVKIVGAGLSVGQVAAVAYGKDASGVTVQLADEA<br>RPRVTTCSEWIVDSVVNGGDIYGVTTGFGGTSHRRTRDGLALQVELVRHLIAGIFGTGREHELPSEVSRASMLVRINTQ<br>LQGYSGIRYEILEAVIKLLNKGVCPRIPLRGSITASGDVVPLAYIAGVLTGRPNAQAVTVDGRIVDAAEAFKIAGIDGGF<br>FKLXPKEGLSIVNGTAVGSALAAMVLFDCNVLAVLSEVLSAVFCEVMHGKPEFADHLIHKLKHHPGSIEAAAIMEHI<br>LAGSSFMVHAKKVNSTDALLKPKQDRYAIRTSPQWLGPQIEVIRAATKCIEREVNSVNDNPVIDLQRGTALHGGNFQ<br>GTPIGVSMDNTRLAIANIGKLMFAQFSELVNEFYNNGLTSNLAGSRNPSLDYGLKGIEIAMAAYCSELQYLANPVTN<br>HVQSAEQHNQGVNSLGLVSARKTAEAVDMLKLMSATYMVALCQAVDLRHLEENIKSYVTKCVAQLAETVLDAPG	691	74.315

			SEEKLTAAIDRVAVFSYADDPCCDHYPLMQKLRSVLLEHALASRSDDEEVLSKIGKLEEELASALPREIEAARRAVEKGTAPVPNLIKGSRSFPVYRFVREELGCVFLTGEKLLGPGEECDKVFVGISQGKHIDPMFECLKEWDGKPLPINIK		
78	P06593	Phytochrome A type 3	MSSSRPASSSSSRNRQSSQARVLAQTTLDAELNAEYEESGDSFDYSKLVEAQRDGPPVQQGRSEKVIAYLQHIQKGKLIQTFGCLLALDEKSFNVIAFSENAPEMLTTVSHAVPSVDDPPRLGIGTNVRSLFSDQGATALHKALGFADVSLLNPILVQCKTSGKPFYAIVHRATGCLVVDFEPVKPTEFPATAAGALQSYKLAAKAISKIQSLPGGSMEVLCNTVVKEVFDLTGYDRVMAYKFHEDDHGEVFSEITKPGLEPYLGLHYPATDIPQAARLLFMKNKVRMICDCRARSIKVIEAEALPFDISLCGSALRAPHSCHLQYMENMNSIASLVMAVVVNENEEDDEAESEQPAQQQKKKKLWGLLVCHHESPRYVPFPLRYACEFLAQVFAVHVNREFELEKQLREKNILKMQTMLSDMLFREASPLTIVSGTPNIMDLVKCDGAALLYGGKVWRLRNAPTESQIHDIAFWLSDVHRDSTGLSTDSLHDAGYPGAAALGDMICGMAVAKINSKDILFWFRSHTAAEIRWGGAKNDPSDMDDSRRMHPRLSFKAFLEVVKMKSLPWSDYEMDAIHSLQLILRGTLNDASKPKREASLDNQIGDLKLDGLAELQAVTSEMVRLMETATVPILAVDGNGLVNGWNQKAAELTGLRVDDAIGRHILTLVEDSSVPVVQRMLYLALQGKEEKEVRFEVKTHGPKRDDGPVILVVNACASRDLHDHVVGVCFVAQDMTVHKLVMDKFTRVEGDYKAIIHNPNPLIPPIFGADEFGWCSEWNAAMTKLTGWNRDEVLDKMLLGEVFDSSNASCPLKNRDAFVSLCVLINSALAGEETEKAPFGFFDRSGKYIECLLSANRKENEGGLITGVFCFIHVASHELQHALQVQQASEQTSLKRLKAFSYMRHAINNPLSGMLYSRKALKNTDLNEEQMKQIHVGDNCHHQINKILADLDQDSITEKSSCLDLEMAEFLLQDVVVAAVSQVLITCQGKGIRISCNLPERFMKQSVYGDGVRLQQILSDFLFISVKFSPVGGSVEISSKLTKNSIGENLHLIDLELRIKHQGLGVPAELMAQMFEEDNKEQSEEGLSLLVSRNLLRLMNGDVRHLREAGVSTFIITAELASAPTAMGQ	1129	124.993
79	Q7M290	Plasma membrane ATPase	WGEQEASILVPGDIVSIKLGDIVPADARIDQSGLTGESLPVTKNPGDEVFSGSTCKTGTLTLNKGIVGMTGDGVNDAPALKTLHGLQAPESTSLNLPNDKELSEIAEQAK	110	11.341
80	A0A023	Plastid acetyl-CoA carboxylase	VPGSPIFIMRLASQSRHLEVQLLCDQYGNVAALHSRDCSVQRRHQKIIEEGPVTVAPRETVKELEQAARRLAKAVGYVGAATVEYLYSMETGEYYFLELNPRLQVEHPVTEWIAEVNLPAAQVAVGMGIPLWQVPEIRRFYGMDNGGGYDIWRKTAALATPFNFDEVDSQWPKGHCVAVRITSENPDDGFKPTGGNVKEISFKSKPNVWGYFSVKSGGGIHEFADSQFGHVFAYGVTRSAAITNMSLALKEIQIRGEIHTNVDYTVDLL	269	39.839
81	I4IY75	Pollen alergen Ave s 5	MAVQKYKVALFLAVALVAAPSASYAADAGYTAATAPASPAGYTVPGTQPKATTEEQKLIENINAGFKEAEAAAATVPAANKYETFVSTFATASNKAFGEALTTGGANTSSKSQLTDKLDAATKLAYDAAQGATPEAKYDAYVATLSEALRIISGTLQVHALKPAAEEVKAIPAGELVVIDKIDAAFKAAATQANAAPANDKFTVFETAFNKAIKDSTGGTYETYKFVPGLEAAVKQAYAATVASVPEVKYAVFETALKKAITAMAEAEKEAEPAAAVTATATTAAGATAGAGAPAAGGYK V	290	30.425
82	P15904	Protochlorophyllide reductase (EC 1.3.1.33) (Fr)	VVVITGASSGLGLAAAKALAETGKWHVVMACRDFLKASKAAKAAGMADGSYTVMHLDLASLDSVRQFVDAFRRAEMPLDVLVCNAAIYRPTARKPTFTAEGVEMSVGVNHLGHFLLARLLLEDLQKSDYPSRRLVIVGSITGNDNTLAGNVPPKANLGDLRGLAGGLTGASGSAMIDGDESFDGAKAYKDSKVCNMLTMQEFHRRYHEDTGITFSSLYPGCIATTGLFREHIPLFRTLFPPFQKFVTKGFVSEAESGKRLAQVVGEPSLTKSGVYWSWNKDSASFENQLSQEASDPEKARKVWELSEKLVGLA	313	33.797
83	B2MUD6	Putative iron phytosiderophore transporter (Fr)	FFFDFSLTYVGAGMICPHIVNISTLLGAILSYGILWPLISKNKGDWYPADVKESSMKSLYGYKAFICIALIMGDGLYHFTKIITITCKGMYRQFSRKHADNREKNVDNTVSLEDLQRDEVFKRGHLPAWIAYSGYAVLSVVAVVTTPIMFRQVKWYYVVIAYVVAPMLGFANSYGTG	177	19.982
84	B2ZRZ5	Putative iron-phytosiderophore transporter (Fr)	QVLVVDYKLVYPSGTATAVLINGFHTTQGDKNSRKQIRGFLKYFGGSFLWSFFQWFYTGGDACGFIQFPTFGLKAWKQTFFFDFSLTYVGAGMICPHIVNISTLLGAILSYGILWPLISKNKGDWYPANVKESSMKSLYGYKAFICIALIMGDGLYHFTKIITITFKGMYRQFTRKRADNREKNVDNTVSLEDLQRDEVFKKGHIPAWMAYSGYAVLSVVAVVTTPIMFRQVKWYYVVIA YVIAPMLGFAN	251	28.563

85	Q6EUD2	Putative resistance protein	FNEVRLTLEILEHVCEDKQEYENISNFNVLQEILMGNIRNKRFLLVLDDMWEDKDRNGWTKLLAPLKSNQVNGCMVLATTRSTSVAKMIGTMDEVRLSGLDEKEFWLFFKGCAFGN	116	13.465
86	Q6EUD5	Putative resistance protein	LARFVYRDQRIRDYFDLQIWVCVSTKFNEVRLTLEILEHVCEDKQEYENISNFNVLQEILMRNIRNKRFLLVLDDMWEDKDRNGWTKLLAPLKSNQVNGCMVLATTRSTSVAKMIGTMDEVRLSGLDEKEFWLFFKGCAFGNENYEGDPSLQSIGKHIAKTLK	163	19.149
87	Q84LJ8	Putative RNA-binding protein (Fr)	TRASTPNGNKKLDAGYREAQEKPSECPVFLFFSVNTSGQFVGIAEMIGPVDFDKTVDYWQQDKWNGCFSIKWHIVKDIPNNILKHITLENNDNKPVTNSRDTQEVKLEQGLQMLKIFKEHVSKTSILDDFAFYENRQKLMQEKRAKQQVLQGQGGDEKEKNAANGNSAAQQQAVSKESAA PVPGGRAERLKIRC	194	21.886
88	Q84V32	Receptor kinase LRK45	MSKLLAIAILLLPLIDHGIDLATAWDDKDFFKYCPPSRCSQHGPEIRFPFCLESINTSSCGCGSRSIRKLACSGQDTILLHPVLGPYNVSAIDYRHSSMKLIPLVDPCMVLQKKLINFRSSPCPQIDDINDNLLVHFYRSTSLVCCSGEFTPSAADRIAGPISCLSNATHFLYFAAGYEDMSLLPLDCKVVPVSDGLYGRIPTYILDDPIVGRYQQSQLFKESAEIILSSAEMTVYWYDGAQNANTMGDTARSTHKGKLHSACLTLTNSTKVPLVAFLSNHIEPTTSSVAVFVVLLLTTVTMIYLSLRTRYSEEIHFKVEMFLQTYGTSQPTRYTFSEVKKIARRFKEKIGQGGYGSVYRGELQNRVPVAVKMLENSRGEGEVFINEVAPIGLIHHANIVRLVGFCSEGTRRALIYEFMPNESLEKYIFSHDPNIFQHLLVPQKLLDIALGIARGMEYLHQGCNQRILHFDIKPHNILLDYNFNPKISDFGLAKLCPRDQSIVTLTAARGTMGYIAPELYSRNFGEISYKSDVYSFGMLVLEMVSGRRNSDPSIESQNKVYLPEWIYEKVINDHELALPAETTEEEKEKVRQLAIVALWCIQWNPRNRPSMTKVVNMLTGSLQNLQMPPKPYVTSENQLMP	641	72.147
89	Q84V35	Receptor kinase ORK10	MGNPGAFRCSVTLQALIVFSLIAVFAADHVQGGDDGCAPFSCGHLQDISSPFRRQGDPLECGVGAYELGCTSSKATIHINTGTYYVTAINYTNSYFRVMDTNFDTNSSCPLPLWNHLPYVESILDSHGFWDLSIRSGYTACFANCSRAVTNNGAYKPVDCLSADNSHVYIWMSGYFDCRVLDLEPYCGYLAMIPFGEEYSSDMQLQLQGASYADIIQLITKGFTIQFPYEEYREPSLSKDMNICLNHSISYFKELISHASFVNWTRAFFWSDMYFYRCVDRLYTFEHTTSPVVAQLIAVPKFLFVYKYLKTRITIDAVEKFLRIQQMIGPTRYAYTDIVALTSHFRDKLGQGGYGSVYKGVLLPGGAHVAVKMLEGNSSCNGEDFISEVSTLGRIHHVNVVRLVGFCSEELRRALVYEYMPHGSLDKYIFSAEKSFSWDKLNEIALGIARGINYLHQGCDMQILHFDIKPHNILLDTNFVPKVADFGLAKLYPRGDSFVPLSAMRGTVGYIAPEMISRSFGVISSKSDVYSFGMLLLEMAGGRRNADPNAAASSQGYYPSWVYDQLTLQGESGEISPVTANMHELEKKLYESRDRPTMSEVMDMLEAGADGLQMPSRPFFCDEGHIHVEDSYHFTSELTTVSEELTVVSEDEE	653	73.213
90	Q9LDR5	Receptor-like kinase extracellular domain	MSKLLAIAVLLLPLMNHGINLAMAWEDQDFFKHCPLSHCSQHGPEIRYPFCLESSNTSLCGCGSRSIRKLACSGQDTILVHPVLGPYNVRAIDYRRSSMKVIPLVDPCLVLQQKLIVSRSSTFPQVDVINDEGSGFDIVDSYFITYATLVSCSREFTPGATDNIVGPVSCLSNTTHFLYFVDSDEYFSVLPFDCKVVPVSDGSGGRQISLYWSEDPKSIYMLHRYPQSFKESAERILRFAETTVYWDHACRQCNYDGCPSCELSGGSCAFSSQSNGAFCMPDPHGYG	287	31.858
91	Q9LL65	Receptor-like kinase extracellular domain RLK5A2	MSKLLAIAVLLLPLMNHGINLAMAWEDQDFFKHCPLSHCSQHGPEIRYPFCLESSNTSLCGCGSRSIRKLACSGQDTILVHPVLGPYNVRAIDYRRSSMKVIPLVDPCLVLQQKLIVSRSSTFPQVDVINDEGSGFDIVDSYFITYATLVSCSREFTPGATDNIVGPVSCLSNTTHFLYFVDSDEYFSVLPFDCKVVPVSDGSGGRQISLYWSEDPKSIYMLHRYPQSLKESAERILRFAETTVYWDHACRQCNYDGCHHVNSVGEAARSAHRVMEHSACLTLMVMA	287	32.076
92	Q946U2	Reverse transcriptase (Fr)	VRKEVFKFLDAGIIYPIADSQWVSLVHCVPKKGGITVVPNEDNELIPQRVVVVYRMCIDFRRINKVTRKDHYPLPFIDQMLERLSKKTHFCFLDGHSGFSQIVVKAQDQEKTTFTCPYGTYDYRRMPFGLCNAPATFQRCMSAIFHGFCEEIVEVFMDDFSVYGTSFDNCLHNLDKFLQRFEETNLVLNWEKCHFMVNEGIVLGHKISERGIEVDRAKIEAIENMPCPRDIKGIRSILGH AGFYSRFIKDFTKV	254	29.497

93	Q946U3	Reverse transcriptase (Fr)	VRKEVFKLMDAGIIYPIADSEWVSHVHCVPKKGGITVVPNDNDELIPQRIVVGYRMCIDFRKVNKVTKKDHYPLPFID QMLERFSKKTHFCFLDGYSGFSQIVVKQQDQEKTTFTCPYGTYAYRCMPFGLCNAPSTFLRCMSAIFHGFCEEIVEVF MDDFSVYGTSFDNCLHNLDKVLQRCEGTNLVLNWEKCHFMVNEGIVLGHKVSKRGIEVDRAKVEAIEKMPCPRDI KGIRSILGH AGFYRRFIKDFTKV	254	29.305
94	Q7HP W6	Ribosomal protein subunit 12s	MPTKNQLIRHGREEKRRTDRTRASDQCPQKQGVCLRVSTRTPKKPNSALRKIAKVRLSNRHDIFAHIPGEGHNSQEH SIVLVRGGRVKDSPGVKSHRIRGVKDLLGIPDRRKGRSKYGAE RPKSK	125	14.197
95	P48684	Ribulose bisphos-phatase carbox-ylaser large chain (RuBisCO large subunit)	MSPQTETKASVGFQAGVKDYKLTYYTPEYETKDTDILAAFRVTPQPGVPPEEAGAAVAAESSTGTWTTVWTDGLTSL DRYKGRCYHIEPVAGEDNQWICYVAYPLDLFEEGSVTNMFTSIVGNVFGFKALRALRLEDLRIPPAYTKTFQGPPHGI QVERDKLNKYGRPLLGCTIKPKLGLSAKNYGRACYECLRGGLDFTKDDENVNSQPFMRWRDRFVFCAEAIYKAQAE TGEIKGHYLNATAGTCEEMIKRAVFARELGVPIVMHDYITGGFTANTSLAHYCRDNGLLLHIHRAMHAVIDRQKNH GMHFRVLAKALRMSGGDHIHSGTVVGKLEGEREMTLGFVDLLRDDFIEKDRARGIFFTQDWVSMPGVIPVASGGIH VWHMPALTEIFGDDSVLQFGGGTLGHPWGNAPGAAANRVALEACVQARNEGRDLAREGNEIIREACKWSPELAA ACEVWKAIKFEFEPVDTIDE	477	52.935
96	L7NW U7	Ribulose bisphos-phate carboxylase large chain	SVGFKAGVKDYKLTYYTPEYETKDTDILAAFRVTPQPGVPPEEAGAAVAAESSTGTWTTVWTDGLTSLDRYKGRCYH IEPVVGEDNQYIAYVAYPLDLFEEGSVTNMFTSIVGNVFGFKALRALRLEDLRIPPTYSKTFQGPPHGIQVERDKLNKY GRPLLGCTIKPKLGLSAKNYGRAC YECL	184	20.405
97	Q2EFG 6	Tasselseed2-like short-chain dehy-drogenase/reduc-tase (Fr)	GARGIGEAIVRLFVKHGAKVVTADIDEAAGEALAASLVPHVAFVRCDVSVEEDVERAVDRAVSRHGRLDVFCNNA GILGRQTCAAKSILSFDAGEFDRVLRVNALGTALGMKHAGRAMMARRYGSIVSVASVAGVLGGLGPHAYTASKHAI VGLTRNAACELGAHGIRVNCVSPFGVATPMLINAWRQGHDASAADDADADIDLDKIAVPSDQEVEKMEEVVRSLA TLKGSTLRPRDIAEAVLFLASDDSRYVS	254	26.67
98	P54411	T-complex protein 1 subunit epsilon	MALDFDEYWRPFIILREQEKKSRLQGLDAQKANIAAGKSVARILRTSLGPKGMDKMLQSPDGDVTITNDGATILELM DVDNQIAKLMVELSRSQDYDIGDGTTGVVVMAGSLLEQAEKLLERGIHPIRVAEGYEMASRIAVDHLESISTKYEFSA TDIEPLVQTCMTTLSSKIVSRCKRALAEIAVKAVLAVADLERKDVNLDLIKVEGKVGGKLEDTELVQGIIVDKDMSHP QMPKRIEDAHIAILTCPFEPPKPKTKHKVDIDTVEKFQTLRGQEQKYFDEMVQKCKDVGATLVICQWGFDDEANHL LMQRELPAVRWVGGVELELIAIATGGRIVPRFQELSTEKLGKAGLVREKSFGTTKDRMLYIEKCANSKAVTIFIRGGNK MMIEETKRSIHDALCVARNLIINNSIVYGGGSAEISCSIAVEAAADRHPGVEQYAIRAFADALDAIPLALAENSGLPPI DTLTVVKSQHVKENNSRCGIDCNDVGTNDMKEQNVFETLIGKQQQILLATQVVKMILKIDDVITPSEY	535	59.034
99	P50695	Thaumatin	MATSSAVLFLLLAVFAAGASAATFRITNNCGFTVWPAGIPVGGGFQLNSKQSSNINVPAGTSAGRIWGRTGCSFNNG RGSCATGDCAGALSCTLSGQPATLAEYTIGGSQDFYDISVIDGFNLAMDFSCSTGVALKCRDANCPDAYHHPNDVA THACNGNSNYQITFCP	169	17.353
100	P50698	Thaumatin	MATSSTVLFLLLAVFAASASAATFTITNNCGYTVWPAAIPVGGGQQLDQGQTWTLNVPAGTNSGRIWGRTGCSFNG GSGSCQTGDCAGALSCTLSGQPATLAEFSIGGEHDYYDISVIDVYNLAMDFSCSTGDALQCRDSSCPDAYHQPDDPK THSCNTNSNYQITFCP	169	17.621
101	Q8LSZ9	Thionin Asthi5	MATTKGLKCVVLCVLVLGLVLGQVQVEGKSCCPSTSARNCYNVCRLTGTSRPRCASLCGCKIVDGTCPDGYSKLHLL LESGEPDVTEYCTIGCMTSVCDNMDNVIHGQEMKIDMLLCNKE CVRFCNKGAVIPSFQA	136	14.608
102	Q8LT00	Thionin Asthi4	MATSKGIKSAVICFLMLGLVLEQVQVEGKSCCKSTTAINCYNVCRLAGAPRPVCAGPCGCKLLDVTTCPSDWPKQH LLSEYGEADAAEYCTIGCMTSVCDNIGNAMFAPIVRGQEMNIDM QVCNNACVRFCNKGAVNPSVGA	142	15.01
103	Q8W1R 3	Transposase (Fr)	IDEKWFYRTRKNPKFYLGLNEEDLKRTTQNKNYIKKVMFLAAVARPRYDDDGNMTFDGKIGIWPFTFLEEAKRDSK HRDVGTIVTKVLPVVTRKVSQDYMVNKLLPANKEKWPASECSYP IIIQQDNA	128	15.047

104	Q8W1R 2	Transposase (Fr)	IDEKWFYMTKKNRNYYLLYGEEEPTRTIQNGSCIGKVMFLTAIARPRWDSEGNVTFSGKIGIWPFVKEVPAQRRSDNR PRGTMETKSIKVNRQVMREFMIDNLLPAIQASWPENDAGQTIYIQQDNA	127	14.808
105	Q8W1R 1	Transposase (Fr)	IDEKWFYRSRLNQKFYLCNDEPDPHRCTKHKSHIDKVMFLCAVARPRFDAAGNCTFDGKLGVWAFVEEVAAKKKS KNRGRGTLETKVLPKVNMQVNRQYLINHVLPAIKAKWPEEDRHQT IWIQQDNA	128	15.024
106	A7U438	Tryptophanin	MKIFFFLALLALVVSATFAQYVESDGSYEEVEGAHDRCQQHQMKLDSCREYVADGCTTMRDFPITWPWKWWKGG CEEVRNECCQLLGQMPSECRCDAIWRSIQHELGGFFGTQQGLIGKR LKIAKSLPTQCNMGPECNIPVTFGYYW	147	16.893
107	A7U440	Tryptophanin	MKALFLLAFLALAASAAFAQQYADTGVGGWDGCMPEKARLNSCKDYVVERCLTLKDIPITWPWKWWKGGCESEV RSQCCMELNQIAPHCRCKAIWRAVQGELGGFLGFQQSEIMKQVHVA QSLPSRCNMGPNCNFPTNLGYY	142	15.901
108	Q38771	Tubulin alfa-chain	MREIISIHIGQAGIQVGNACWELYCLEHGIQQDGTMPSDTTVGVAHDAFNTFFSETGAGKHVPRAIFVDLEPTVIDEV RTGAYRQLFHPEQLISGKEDAANNFARGHYTVGKEIVDLCLGRVRQLADNCTGLQGFLVFNAVGGGTGSGLGSLLL ERLSVDYGKKSKLGFTIYPSPQVSTAVVEPYNSVLSTHSLLEHTDVAVLLDNEAIYDICRRSLDIERPTYTNLNRLISQIIS SLTTSLRFDGAINVDVTEFQTNLVPYPRIHFMLSSYAPVISAEKAYHEQLSVPEITNAVFEPSSMMAKCDPRHGKYMA CCLMYRGDVVPKDVNAAVVTIKTKRTVQFVDXCPTGFKCGINYQPPSVVPGGDLAKVQRAVCMISNNTAVAEVFSR IDHKFDLMYAKRAFVHWYVGEGMEEGEFSEAREDLAXLEKXYEEVGAEGADDEVTRG	447	49.204
109	P25862	Tubulin beta-1 chain	AVLMDLEPGTMDSVRTGPYGQIFRPDNFVFGQSGAGNNWAKGHYTEGAELIDSVLDVVRKEAENCDCLQGFQVCH SLGGGTGSGMGTLLISKIREEYPDRMMLTFSVFPSPKVSDTVVEPYNATLSVHQLVENADECMVLDNEALYDICFRTL KLTTPSFGDLNHLISATMSGVTCCLRFPGQLNSDLRKLAVNLIPFPRLHFFMVGFAPLTSRGSQQYRALTVPELTQQM WDSKNMMCAADPRHGRYLTASAMFRGKMSTKEVDEQMINVQNKNSSYFVEWIPNNVKSSVCDIPPTGLSMASTFI GNSTSIQEMFRRVSEQFTAMFRRKAFLHWYTGEGMDEMEFTEAESNMNDLVSEYQQYQDATADEEGEYEDEEEDLQ AEDM	386	43.354
110	O22678	UDP-glucose:sterol glucosyltransferase	MADAEPTGGGGKGAEDIGGAAEAHSRDSPASAALPTAPSTSSSSADNGNLHRSSTMPGVIKDAEIITETTGPSNFERS KTERRRQNNDPAKQLLDDKISVRKKLKMLNRIATVRDDGTVVVDVPSSLDLAPLDVGGEDGYGDVTVEESLDGADI PSIPPMQIVILIVGTRGDVQPFVAIAKRLQDYGHRVRLATHANYKEFVLTAGLEFFPLGGDPKLLAEYMVKNKGFLPS GPSEIPIQRKQMKEIIFSLLPACKDPDPDTGIPFKVDAIIANPPAYGHTHVAEALKVPIHIFFTMPWTPTSEFPHPLSRVK TSAGYRLSYQIVDSMIWLGIRDMINEFRKKKLKLRPVTYLSGSQGSGSDIPHGYIWSPHLVPKPKDWGPKIDVVGFCFL DLASDYEPPEELVKWLEAGDKPIYVGFGSLPVQDPTKMTETIIQALEMTGQRGIINKGWGGLGTLAEPKDSIYVLDNC PHDWLFLQCKAVVHHGGAGTTAAGLKAACPTTIVPFFGDQQFWGDRVHARGVGPVPIPVEQFNLQKLVDAMKFM LEPEVKEKAVELAKAMESEDGVTGAVRAFLKHLPSSKEDENSPPPTPHGFLEFLGPVSKCLGCS	608	65.946
111	G3GH M2	Vacuolar H^+-ATPase subunit H (Fr)	LTSIHDVLKGLVDWLCSQXRSPTHPNCSIPTATHCLATLLKETYVRTLFVQADGVKLLIPLISPASTQZSIQLLYETCLCI WLLSFYDAAVDYLSTTRVIPRLVEVVKGSTKEKXVRVVVMSFRNLLAKGAFAAQMIDLGLPHIVQNLKAQAWSDED LLDALNQLEVGLKENLKRXSSFDKYKQQVLLGHLDWSPMHKDPNFWXENITXFEGNEFQIXRVLMTVIDTSTDTHA LAVACYDLSQXLQYHPXGRLXXADLKAXXRVMXLXNHDNAEVRKNSLLCVQRLFLGAKYXSFLQA	298	33.641
112	Q38766	Victorin binding protein	MERARRLANRALLRRLLAGSASTTTPSPSRGISTLVPSPAAGSRPRRARPAHQHTPSRPVSVSALQPSDTFPRRHNSAS PAEQTVMASTCGFNTLDSLIDATVPAAIRAPPMQFTGKFDAGFTESQMLEHMAHLASMNKVYKSFIGMGYYNTHIP AVILRNLMENPAWYTQYTPYQAEIAQGRLESLLNYQTMVADLTGLPMSNASLLDEATAAAEAMAMCNGILKAKKK TFLIASNCHPQTIDICQTRAAGFDLNVVVSDAKDFDYSSGDVCGVLVQYPGTEGEVLDYAEFVKDAHKHGVKVVMA TDLLALTTLRPPGEIGADIAVGSAQRFGVPMGYGGPHAAFLATSQEYKRLMPGRIIGVSVDSSGKPALRMAMQTREQ HIRRDKATSNICTAQALLANMAAMYAVYHGPAGLKAIADRVHGLAGTFAHGLKKLGTVTVQELPYFDTVKITCAD ANAIAEEARKNEMNLRVVDANTITVAFDETTTLEDVDKLFKVFSGGKPVDFTAESIAPEVSSSIPSSLVRDSPYLTHPIF	1032	111.4

			SMYHTEHELLRYLHKLQTKDLSLCHSMIPLGSCTMKLNATVEMMPVTDPKFANMHPFAPIDQAAGYHEMFDNLGE LLNTITGFDSFSLQPNAGASGEYAGLMVIRAYHRARGDHHRNVCIIPVSAHGTNPASAAMCGMKIITVGTDSKGNIN IPELKKAAEANKDNLSALMVTYPSTHGVYEEGIDEICRIIHDNGGQVYMDGANMNAQVGLTSPGFIGADVCHLNLH KTFCIPHGGGGPGMGPIGVKKHLAPFLPSHPVIPTGGFPLPEKTDPLGSISAAPWGSALILPISYTYIAMMGSQGLTDAS KIAILNANYMAKRLEKHYPVLFRGVNGTVAHEFIIDLRGFKATAGIEPEDVAKRLMDYGFHGPTMSWPVPGTLMIEP TESESKAELDRFCDALISIREEIAQVENGIADVNNNVLKGAPHPPQLLMSDAWTKPYSREYAAFPAAWLRGAKFWPT TCRVDNVYGDRNLICTLQQA SQVAEEAAAATA		
113	R4I4D6	Vromindoline 1.1	MKTLLLLALLALAASTAFAQYAQDDGWNEQGGEATGCEQQQANLDSCKDYVTERCFTMKDFPLTWPWKWWKG GCEHEVRYQCCEQLNQVSQQCRCKAIWRAVEHELGGFLGLQKGEIGKRLLRAKSIPSKCNMGPQCNFPLTTGYYW	147	16.765
114	R4I3I8	Vromindoline 3	MKALFLLAFLALAASAAFAQQYANTRAGGWDGCMPEKARLNSCKDYVVERCLTLKDIPITWPWKWWKGGCESEV RGQCCMELNQIAPHCRCKAIWRAVQGELGGFLGFQQSEIMKQVHVA QTLPSKCNMGPNCNFPTNLGYY	142	15.928
115	P23957	V-type 165 proton ATPase 16 kDa proteolipid subunit	MSSVFSGDETAPFFGFLGAAAALVFSCMGAAYGTAKSGVGVASMGVMRPELVMKSIVPVVMAGVLGIYGLIIAVIIST GINPKAKPYFLFDGYAHLSSGLACGLAGLAAGMAIGIVGDAGVRANAQQPKLFVGMILILIFAEALALYGLIVGIILSS RAGQSRAD	165	16.621

Fr – Fragment of protein sequence

Table S2. Peptides with antioxidant activity and cardiometabolic syndrome (CMS) key enzymes inhibition activity encrypted in oat protein sequences released using *in silico* digestion and their overall solubility. Data obtained using the BIOPEP-UWM database [77] (accessed March-April 2020).

**Oat proteins antioxidative peptides and CMS enzyme inhibitors**					
CF*	GQ	IPA*	NE	PR	TW*
DA	GR	IPY*	NF*	PSY*	TY*
DG	GTW*	IQ*	NHK	PW*	VA*
DQ	GW*	IR	NK	PY*	VE
DR	GY*	IVR	NL*	SF*	VF*
EL	HA*	IVY*	NQ	SK	VGL*
GA*	HE	IW*	NR	SL*	VIY*
GE	HF*	IY*	NW*	SW*	VK
GF*	HIR	MA*	NY*	SY*	VL*
GGE	HK	ME	PA*	TA*	VP*
GGY*	HL*	MF*	PF*	TDY	VPK
GHF*	HR	MK	PGL*	TE	VPL*
GI*	HW*	ML*	PHA*	TF*	VQ*
GK	HY*	MQ*	PK	TK	VR
GL*	IA*	MR	PL*	TL*	VS
GPA*	IE	MW*	PP*	TQ	VW*
GPGE	IF*	MY*	PPL*	TR	VY*
GPL*	IL*	NA	PQ	TT*	

* poor solubility

**Solubility**	**% of peptides**
poor	60.75
good	39.25

Minkiewicz, P.; Iwaniak, A.; Darewicz, M. BIOPEP-UWM database of bioactive peptides: Current opportunities. Int. J. Mol. Sci. 2019, 20, 5978, doi:10.3390/ijms20235978.

Table S3. Peptides identification in oat kernel digests

Peptide	Molecular formula	*m/z*	z	R_t [min]	MS/MS fragments	Precursor protein
GF	C11H14N2O3	223.10776	1	2.53	91.05; 120.08; 131.05; 149.06; 166.09; 177.10	globulins, other proteins
HF	C15H18N4O3	303.14521	1	1.27	93.04; 110.07; 138.07; 166.06; 257.14; 285.13	globulins, other proteins
HW	C17H19N5O3	342.15611	1	1.73	110.07; 138.07; 188.07; 205.10; 296.15; 324.14	other proteins
IF	C15H22N2O3	279.17036	1	6.16	69.07; 86.10; 166.09; 233.16	other proteins
ML	C11H22N2O3S	263.14244	1	3.55	86.10; 104.05; 198.11	globulins, other proteins
PL	C11H20N2O3	229.15471	1	1.99	70.06; 86.10; 132.10; 183.15	globulins, other proteins
PR	C11H21N5O3	272.17176	1	1.74	70.07; 112.09; 175.12; 212.14; 255.14	globulins, other proteins
PW	C16H19N3O3	302.14996	1	4.27	70.07; 188.07; 205.10; 256.14	Other proteins
SF	C12H16N2O4	253.12001	1	2.50	91.05; 144.08; 162.09; 166.09; 235.11	globulins, other proteins
TF	C13H18N2O4	267.13398	1	2.86	91.06; 120.08; 249.12	globulins, other proteins
TW	C15H19N3O4	306.14488	1	3.97	84.04; 130.07; 132.08; 159.09; 170.06; 171.09; 289.13	other proteins
VF	C14H20N2O3	265.15471	1	4.25	55.05; 72.08; 120.08; 166.09; 219.15	globulins, other proteins
VW	C16H21N3O3	304.16561	1	5.15	55.05; 56.04; 72.08; 73.09; 83.05; 118.07. 132.08; 159.09; 187.08; 188.07; 189.07; 205.10; 206.10; 215.07	other proteins

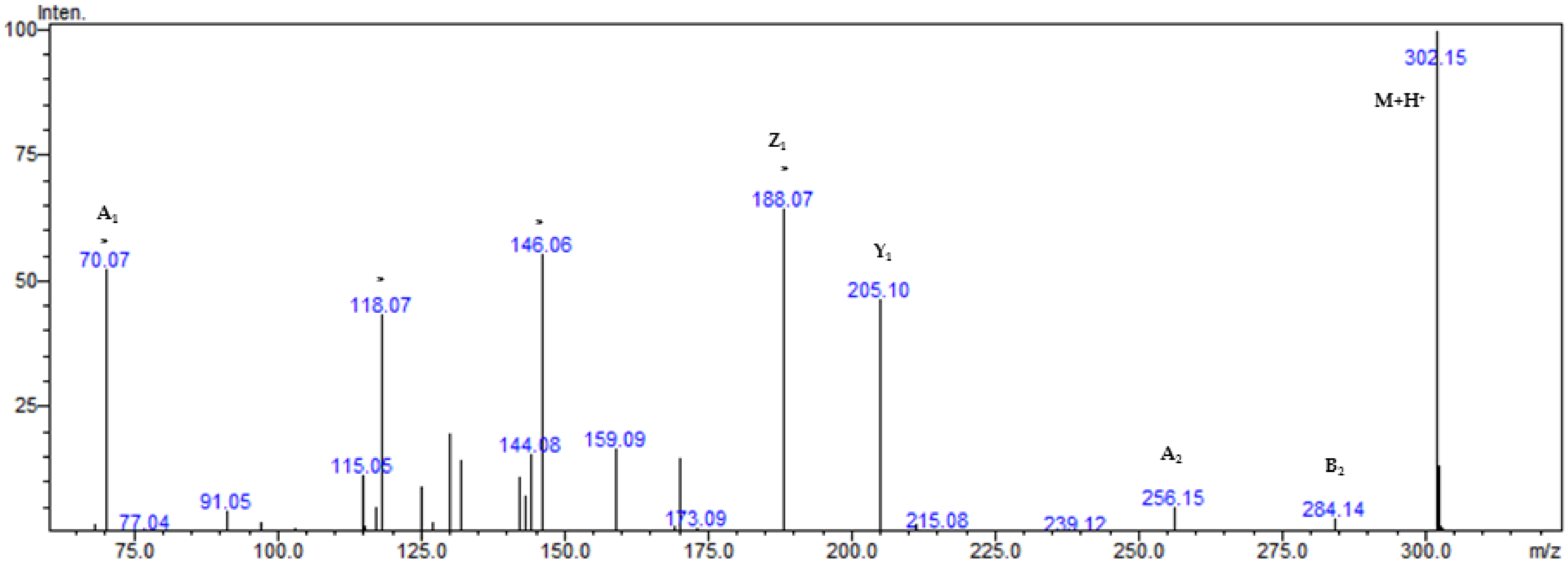

Figure S1. MS/MS spectra of fragment ions obtained from the precursor ion with *m/z*=302.15 from the "gastrointestinal" hydrolysate after *in vitro* digestion of oat kernel proteins (after Caco-2 experiment) obtained during the identification of the PW sequence peptide using the Q-TOF mass spectrometer. *mass of fragmentation ions appearing also on METLIN spectra. Fragment ion nomenclature according to Roepstorff and Fohlman (1984) [60]

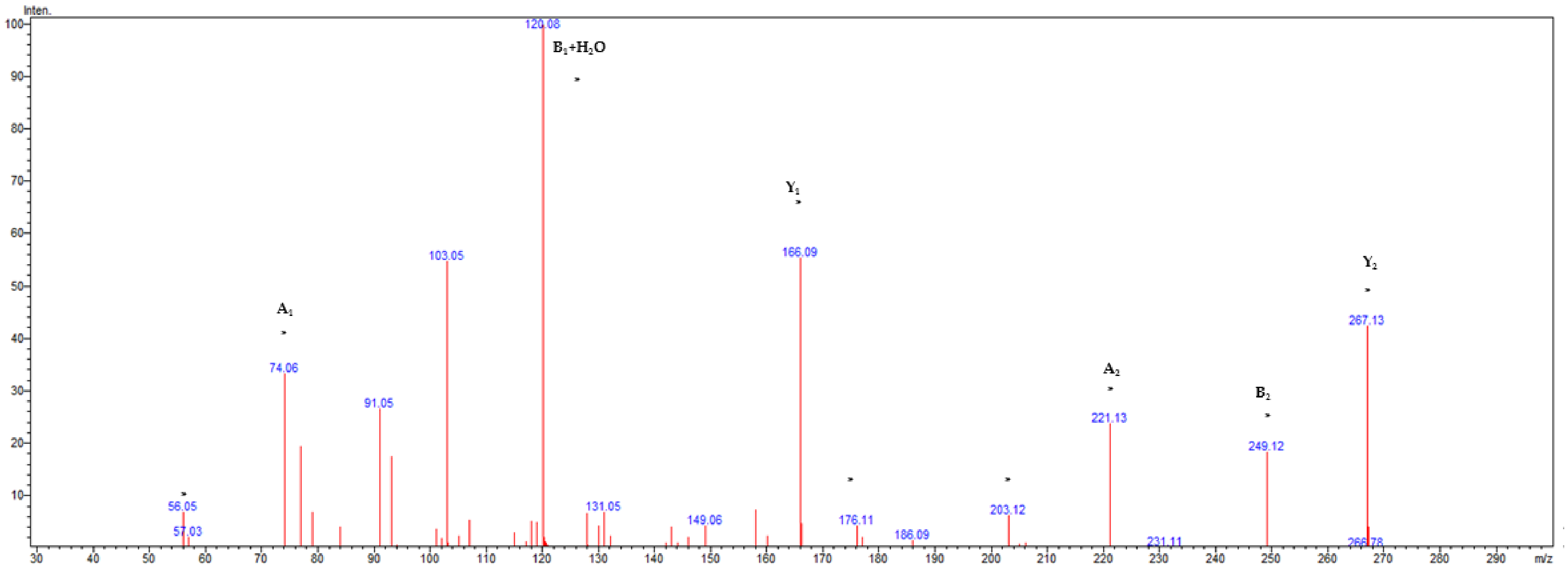

Figure S2. MS/MS spectra of fragment ions obtained from the precursor ion with m/z=267.13 from the "gastrointestinal" hydrolysate after *in vitro* digestion of oat kernel proteins (after Caco-2 experiment) obtained during the identification of the TF sequence peptide using the Q-TOF mass spectrometer. *mass of fragmentation ions appearing also on METLIN spectra.

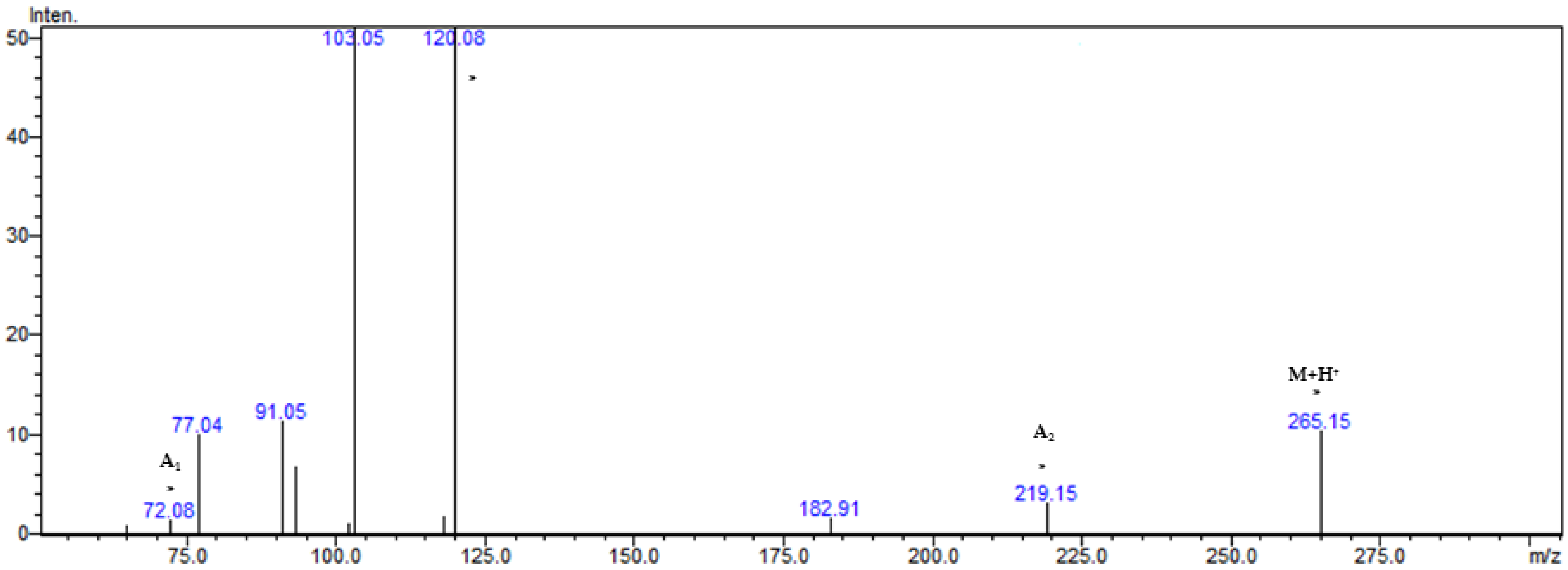

Figure S3. MS/MS spectra of fragment ions obtained from the precursor ion with *m/z*=265.15 from the "gastrointestinal" hydrolysate after *in vitro* digestion of oat kernel proteins (after Caco-2 experiment) obtained during the identification of the VF sequence peptide using the Q-TOF mass spectrometer. *mass of fragmentation ions appearing also on METLIN spectra.

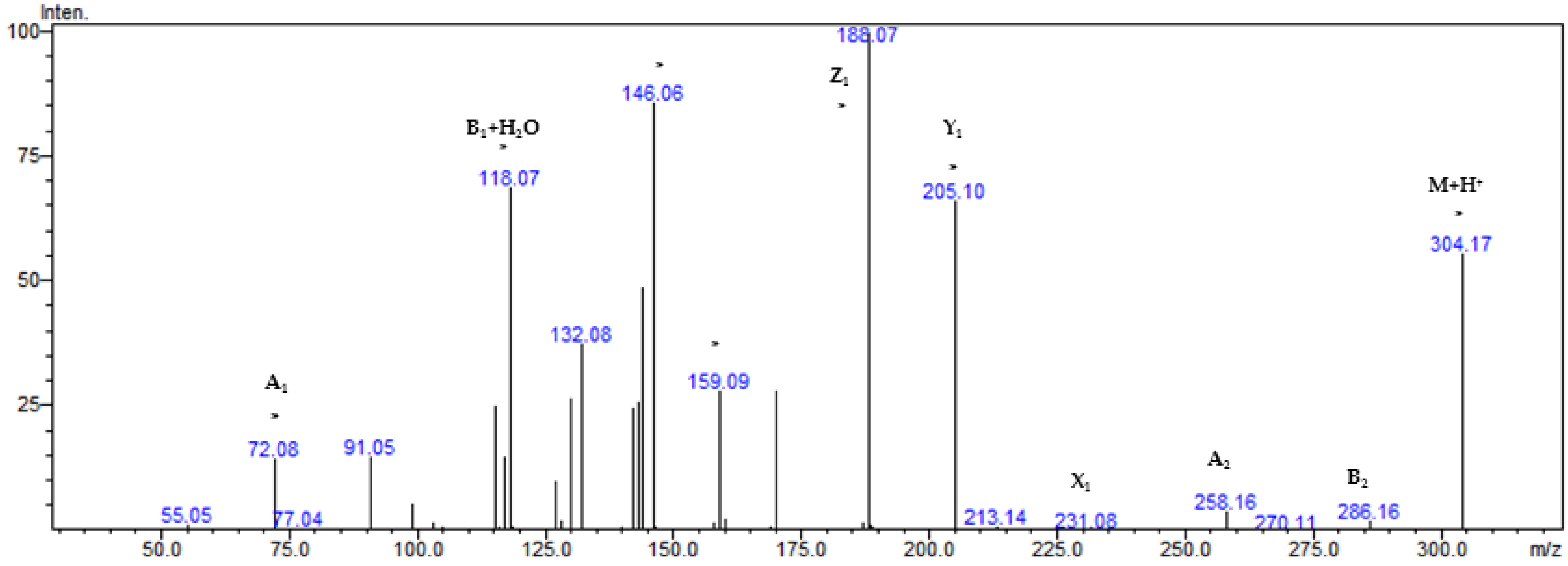

Figure S4. MS/MS spectra of fragment ions obtained from the precursor ion with *m/z*=304.17 from the "gastrointestinal" hydrolysate after *in vitro* digestion of oat kernel proteins (after Caco-2 experiment) obtained during the identification of the VW sequence peptide using the Q-TOF mass spectrometer. *mass of fragmentation ions appearing also on METLIN spectra.
